# β^2^-*Homo*-Amino Acid Scan of µ-Selective Opioid Tetrapeptide TAPP

**DOI:** 10.3390/molecules25102461

**Published:** 2020-05-25

**Authors:** Dagmara Tymecka, Piotr F. J. Lipiński, Piotr Kosson, Aleksandra Misicka

**Affiliations:** 1 Faculty of Chemistry, University of Warsaw, Pasteura 1, 02–093 Warsaw, Poland; 2 Department of Neuropeptides, Mossakowski Medical Research Centre Polish Academy of Sciences, Pawińskiego 5, 02–106 Warsaw, Poland; plipinski@imdik.pan.pl; 3 Toxicology Research Laboratory, Mossakowski Medical Research Centre Polish Academy of Sciences, Pawińskiego 5, 02–106 Warsaw, Poland; pkosson@imdik.pan.pl

**Keywords:** β^2^-amino acids, β^2^-*Homo*-amino acids, µ-opioid receptor, opioid peptides, TAPP, racemic synthesis of β^2^-amino acids

## Abstract

TAPP (H-Tyr-d-Ala-Phe-Phe-NH_2_) is a potent, µ-selective opioid ligand. In order to gain further insights into pharmacophoric features of this tetrapeptide, we have performed a β^2^-*Homo*-amino acid (β^2^hAA) scan of the TAPP sequence. To this aim, 10 novel analogues have been synthesized and evaluated for µ-opioid and δ-opioid receptor affinity as well as for stability in human plasma. The derivatives included compounds in which a (*R*)- or (*S*)-β^2^-*Homo*-Homologue replaced the amino acids in the TAPP sequence. The derivatives with (*R*)- or (*S*)-β^2^hPhe^4^ turned out to bind µOR with affinities equal to that of the parent. β^2^hAAs in position 1 and 3 resulted in rather large affinity decreases, but the change differed depending on the stereochemistry. β^2^-*Homo*logation in the second position gave derivatives with very poor µOR binding. According to molecular modelling, the presented α/β-peptides adopt a variety of binding poses with their common element being an ionic interaction between a protonable amine of the first residue and Asp147. A feature required for high µOR affinity seems the ability to accommodate the ring in the fourth residue in a manner similar to that found for TAPP. Contrary to what might be expected, several compounds were significantly less stable in human plasma than the parent compound.

## 1. Introduction

**Figure 1:**
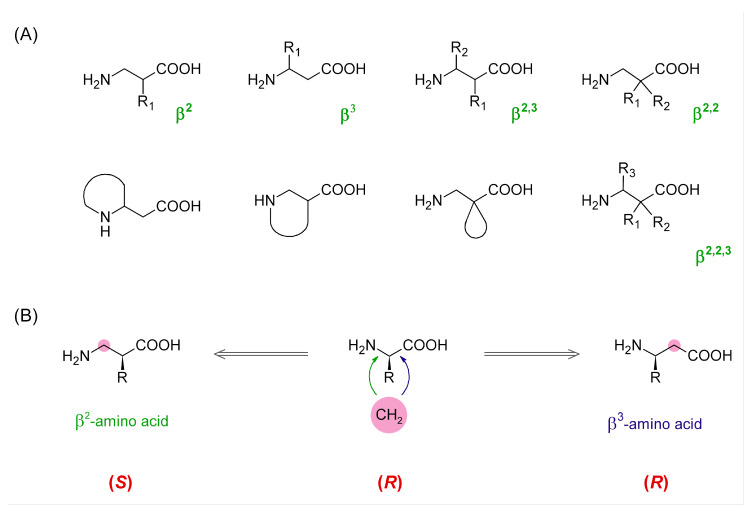
(**A**) Examples of diverse possible types of β-amino acids. (**B**) Comparison of absolute configuration of α-, β^2^-, and β^3^-amino acids.

There is a rich repertoire of structural modifications that a medicinal chemist can use when exploring structure-activity relationships of peptide active compounds or working on the improvement of their physicochemical properties. This repertoire includes, e.g., backbone cyclization, introduction of peptide bond isosteres, incorporation of d-amino acids, α,α-disubstituted amino acids, residues with substituted or constrained side-chains, and other unnatural amino acids [1]. In the latter group, a prominent place is occupied by β-amino acids (β-AAs) [2,3]. The amino acids of this class have two carbon atoms between the carboxylic and amino groups. With both mono-substitutions and poly-substitutions being possible in different combinations and stereochemistries (Figure 1), β-AAs constitute a family of building blocks of enormous structural diversity.

β-Amino acids can be used to construct full β-peptides (solely made of β-AAs), but it is also possible to create mixed α/β-peptides in which one or more β-residues are incorporated instead of some α-amino acids. There are three main structural consequences of introducing a β-residue into a peptide backbone. First, each β-AA elongates the backbone by one methylene unit. Furthermore, it adds one freely rotating sp^3^-sp^3^ C-C bond, which increases the backbone flexibility. Lastly, in topographical terms, the position of side-chains of the residues that follow a β-residue is shifted toward the C-terminus. All these changes can have critical impact on activity and other properties (e.g., stability) of a peptide sequence and this is why β-AAs are useful means in medicinal chemistry research.

Peptides are endogenous ligands for all types of opioid receptors (µ, δ, κ, and nociceptin) [4]. Among these receptors, the µ type (µ-opioid receptor, µOR) constitutes a major molecular target for the treatment of pain, but the medicinal potential of the remaining ORs has been increasingly acknowledged [5]. Over the years, both natural and synthetic peptides as well as peptide-inspired organic analogues have continued to be key compounds in the research on the opioid receptors.

In attempts to decipher structure-activity relationships of opioid peptides, or in search for more stable analogues, the researchers also used β-AAs. All-β analogues (that is with β^3^-*Homo*-AA in every position) of deltorphin I, Leu-enkephalin, and dermorphin were reported by Wilczyńska et al. [6]. The same paper presented several α/β-hybrides of deltorphin I. Alicyclic β-amino acids as proline mimics were used for studying conformational requirements in endomorphin-1/-2 analogues [7,8]. Other authors replaced proline for β-alanine [9]. Earlier, cyclic β-AAs were applied in studies on morphiceptin [10,11] and dermorphin [12]. Cardillo et al. conducted a systematic β-AA [13] and β^3^-*Homo*-AA scan of endomorphin-1 [14], exchanging each single AA in the sequence for its β-isomer or β^3^-Homologue. In another work, β^3^-*Homo* and β^2^-*Homo* aromatic amino acids were introduced alone or in combination in positions 3 or/and 4 of endomorphin sequence [15,16]. Mollica et al. performed a β^3^-*Homo*-AA scan of a dimeric peptide, biphalin [17]. Several derivatives of this peptide with β^3^hPhe or β^3^-*Homo*-p-NO_2_-Phe were reported by Frączak et al. [18]. Dimeric derivatives of dermorphin were probed with β^3^-hAAs as well [19]. Recently, Adamska-Bartłomiejczyk et al. incorporated β-AAs into the structure of a cyclic endomorphin derivative, Tyr-c[D-Lys-Phe-Phe-Asp]NH_2_ [20]. It is also polysubstituted β-AAs that were used in the opioid field. For example, several recent papers dealt with endomorphin-1/-2 analogues containing one or more β^2,3^-AAs [21,22,23,24,25].

An interesting µOR selective opioid peptide is TAPP (H-Tyr-d-Ala-Phe-Phe-NH_2_, **1**) [26]. Structurally, it resembles endomorphin-2 (H-Tyr-Pro-Phe-Phe-NH_2_) in having Phe-Phe in positions 3 and 4, but it is also similar to some enkephalin-based derivatives, such as DAMGO (H-Tyr-d-Ala-Gly-*N*-MePhe-Gly-ol), in having d-Ala in the second position. The TAPP sequence was probed by a β^3^-*Homo*-AA scan by Podwysocka et al. [27]. In the presented research, we wanted to complement their work by conducting a β^2^-*Homo*-AA scan. To this aim, we have synthesized, tested for receptor affinity, and plasma stability as well as subjected to molecular modelling 10 novel TAPP analogues. In these, α-AAs in each position were replaced by their β^2^-Homologues. Additionally, β^2^h-*m*-Tyr was used in the first position. As we employed both (*R*)-isomers and (*S*)-isomers of β^2^h-AAs, we were able to probe the effect of backbone expansion with the concomitant retention or reversion of side-chain spatial positioning. In this case, it is worth noting that, in β^2^-AAs, the very same spatial arrangement of substituents at the asymmetric carbon as in α-AAs or β^3^-AAs gives usually different absolute configurations (Figure 1B). In addition, a direct β^2^-*Homo*-counterpart (in terms of side-chain location) of (*S*)-Phe is (*R*)-β^2^hPhe (exceptions are β^2^hCys, β^2^hThr, and β^2^hSer).

## 2. Results and Discussion

### 2.1. Chemistry

**Scheme 1:**
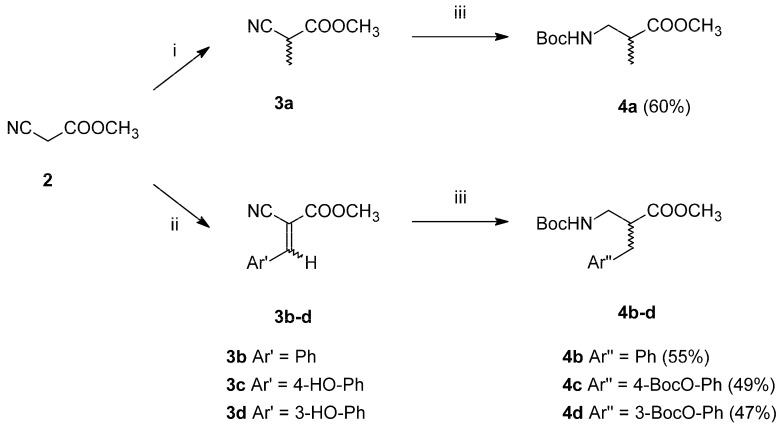
Synthesis of racemic β^2^-*Homo*-amino acids. Reagents and conditions (i) MeI, K_2_CO_3(dry)_, -*N,N* dimethylformamide (DMF), (ii) Ar’CHO, piperidine, MeOH, (iii) CoCl_2_·6H_2_O, NaBH_4_, Boc_2_O, MeOH_(dry)_.

The planned modifications of the TAPP structure required preparation of β^2^-*Homo*-amino acids. Since chiral synthesis of β^2^-amino acids is labour-intensive (several stages of synthesis), we decided to start with β^2^-amino acids in the racemic form. These were prepared in two-step syntheses (Scheme 1), starting from methyl cyanoacetate (**2**). This substrate was either alkylated with methyl iodide to form methyl α-methyl cyanoacetate (**3a**, alanine analogue path) or transformed into Z/E isomers of methyl α-cyano-cinnamates (**3b**–**d**) via Knoevenagel condensation with aromatic aldehydes (aromatic analogues path). In the second step, the intermediates **3a**–**d** were subject to simultaneous one-pot reduction of nitrile groups and double bonds along with Boc-protection. This was possible due to using CoCl_2_-NaBH_4_ combination in anhydrous methanol with di-tert-butyl dicarbonate as a trapping (to prevent dimerization into secondary amines) and a protective agent [28,29]. Thus, fully *N*-protected and *C*-protected racemic β^2^-*Homo*-amino acids (**4a**–**d**) were obtained in moderately good yields. 

**Scheme 2:**
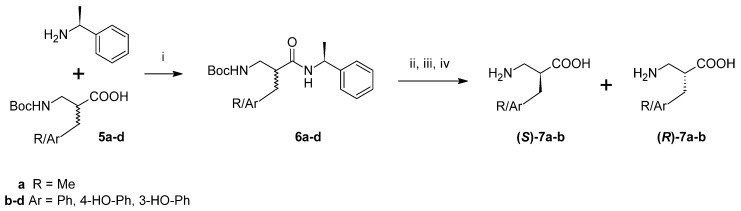
Separation of racemic β^2^-*Homo*-amino acids. Reagents and conditions (i) coupling reagent TBTU, diisopropylethylamine (DIPEA), DMF, (ii) column chromatography: hexane (or CHCl_3_)/AcOEt, (iii) 6M HCl reflux, (iv) ion exchange chromatography (Dowex 50W-X12), NH_3_ aq.

Furthermore, an attempt was made to separate pure enantiomers (Scheme 2). After C-deprotection, the acids (**5a**–**d**) were derivatized by coupling with (*S*)-(−)-α-methylbenzylamine. In the case of alanine and phenylalanine analogues, the resulting diastereoisomeric amide pairs (**6a**–**b**) were separated with column chromatography and further hydrolysed with 6M HCl. After purification (ion-exchange chromatography), the absolute configuration of the products (**7a**–**b**) was determined by measuring specific rotation and comparing it to the literature values. Regarding diastereoisomeric pairs of amides of β^2^h-Tyr and β^2^h-*m*-Tyr (**6c**–**d**), it was possible to separate them using preparative high performance liquid chromatography (HPLC). However, hydrolysis and isolation of the expected products failed.

The planned α/β-tetrapeptides (**8**–**17**) were then synthesized in solution. First, dipeptides were formed from respective α- or β^2^-*Homo* amino acid derivatives. Then, the dipeptides were coupled to yield the tetrapeptides, which was followed by ammonolysis to obtain the desired tetrapeptide amides. In the case of analogues containing β^2^hTyr and β^2^h-*m*-Tyr, diastereoisomeric mixtures were used for dipeptide syntheses and, therefore, diastereoisomeric mixtures of tetrapeptides were obtained. It was possible to separate single isomers by preparative HPLC. The absolute configurations of these derivatives were assigned based on HPLC retention times, per analogiam to isomers of β^2^hPhe-d-Ala-Phe-Phe-NH_2_ (**18**–**19**), on the assumption that the presence of meta-phenol or para-phenol groups does not influence the elution order of diastereoisomers. The names of the analogues for which such an assignment procedure was performed will be further on marked with an asterisk: [(*R*)-β^2^hTyr]-TAPP*, [(*S*)-β^2^hTyr]-TAPP* etc.

### 2.2. Opioid Receptor Affinity

The parent compound (TAPP, **1**) and the synthesized α/β-peptides (**8**–**17**) were tested for binding affinity to μ-opioid and δ-opioid receptors. The determinations were performed by a competitive radioligand displacement assay with selective radioligands. The results are presented in Table 1 (μOR) and Table SM-BIN-1 in the Supplementary Materials (δOR) as half-maximal inhibitory concentration (IC_50_) with standard errors of the mean (S.E.M).

The parent peptide (TAPP) was found to have high affinity for μOR (IC_50_ = 5.1 ± 3.5 nM) and only low binding to δOR, which is consistent with the data in the original TAPP report by Schiller et al. [30]. Introduction of an additional -CH_2_- unit into the TAPP backbone resulted in diverse changes in μOR affinity, depending on the position where the backbone was expanded. Position 4 does not seem sensitive for this modification at all. Derivative [(*R*)-β^2^hPhe^4^]-TAPP (**12**) exhibited IC_50_ of 1.9 ± 2.4 nM, which is equal to (not significantly different than) the value found for TAPP. In position 3, the additional methylene ([(*R*)-β^2^hPhe^3^]-TAPP**, 11**) brought about a more than 10-fold decrease in μOR binding. An even greater drop in affinity was observed for a derivative with the expansion in position 2. For this compound ([(*S*)-β^2^hAla^2^]-TAPP**, 10**) the IC_50_ value was greater than 1000 nM, which means that it is at least a 200-times weaker ligand than the parent. The backbone expansion in the first position decreased the affinity about 15-times ([(*R*)-β^2^hTyr^1^]-TAPP*, **8**). However, in this case, the binding strength may be partially restored if the expansion is accompanied by meta-positioning of the phenol group ([(*R*)-β^2^h-*m*-Tyr^1^]-TAPP*, **9**).

If simultaneously with the introduction of a methylene unit into the backbone, side-chain positioning was inverted, the observed trends in affinity were not parallel to those in the former series. Position four is the least sensitive one, and the derivative [(*S*)-β^2^hPhe^4^]-TAPP (**17**) exhibited similar μOR binding strength as TAPP. Not much worse was the analogue with (*S*)-β^2^hPhe in position 3 (**16**), which is in contrast to the former series where (*R*)-β^2^hPhe in this position (**16**) brought a more pronounced decrease in affinity. Introduction of (*R*)-β^2^hAla in position 2 produced a derivative with very low affinity (**15**, IC_50_ = 954.9 ± 2.9 nM). With respect to position 1, backbone expansion accompanied with an inversion of the side-chain positioning was associated with a large decline in μOR affinity ([(*S*)-β^2^hTyr^1^]-TAPP*, **13**, IC_50_ = 338.8 ± 5.1 nM). Notably, if additionally the phenol group was switched to the *meta*-position, the binding strength was restored and equal to that of the parent peptide ([(*S*)-β^2^h-*m*-Tyr^1^]-TAPP*, **14**, IC_50_ = 11.2 ± 6.3 nM).

As to the δOR affinity (Table SM-BIN-1), derivatives with modifications in positions 1 and 2 (**8**, **9**, **10**, **13**, **14**, **15**) did not exhibit any measurable binding for the δ opioid receptor. Expansion of the backbone in the fourth position accompanied by inversion of the side chain location ([(*S*)-β^2^hPhe^4^]-TAPP, **17**) brought about a slight improvement in δOR affinity when compared to the parent peptide, but the IC_50_ value reads in the high-middle nanomolar range. The remaining three derivatives (**11**, **12**, **16**) exhibited δOR binding at a level similar to the parent TAPP.

Comparing these results to the work by Podwysocka et al. [27], a disparity of trends is noticed. β^3^-*Homo*-AAs in position 1 and 3 gave completely inactive derivatives, irrespectively of the stereochemistry, while, in our work, β^2^-*Homo*-AAs in these positions produced smaller µOR affinity decreases that varied with stereochemistry and [(*S*)-β^2^hPhe^3^]-TAPP can be considered as only a slightly worse binder than the parent compound. On the contrary, β^3^-*Homo*logation in position 2 gave a derivative with nanomolar affinity, and, in our work, analogues with β^2^hAla^2^ did not have appreciable µOR binding. Furthermore, in our report, derivatives with β^3^h-AA in the fourth position are essentially equipotent to the parent, but, for β^2^-hAA^4^ analogues, an affinity decrease by about six times was reported.

### 2.3. Molecular Modelling

In order to understand the observed structure-activity trends in terms of ligand-receptor interactions, the compounds **1** and **8–17** were docked into the µ-opioid receptor structure (PDB accession code: 6DDF [31]) using AutoDock 4.2.6 [32].

**Figure 2:**
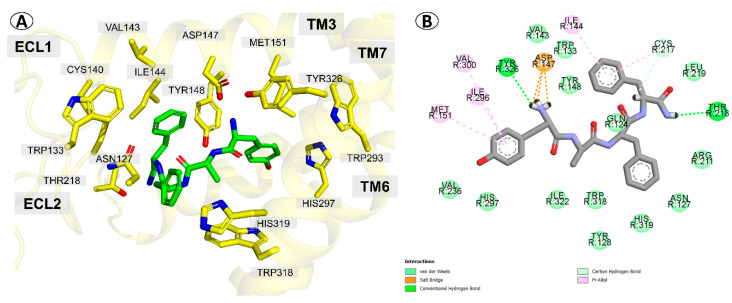
µOR binding mode of the parent compound (TAPP, **1**) as predicted by docking. (**A**) The peptide (green sticks) in the binding site of the receptor (yellow). Side chains of only several residues are shown. (**B**) The interactions scheme.

The best scored pose for the parent TAPP (**1**) is presented in Figure 2A. Docking predicts that TAPP binds µOR with the N-terminal Tyr^1^ directed toward the intracellular part of the receptor. The complex is stabilized by a canonical ionic interaction of protonated Tyr^1^ amine with Asp147. The phenol group of this residue is involved in hydrogen bonding with His297. Furthermore, the aromatic ring forms several dispersive (π-alkyl) contacts with side chains of Met151, Ile296, and Val300. At the C-terminus, the terminal amide interacts with Thr218 via the hydrogen bond. The Phe^4^ is located in a hydrophobic subsite formed by several residues of transmembrane helix 3 (TM3) and extracellular loops 1 and 2 (ECL1 and ECL2). There the aromatic ring participates in dispersive (π-alkyl) interactions with Trp133, Ile144, and Cys217. The Phe^3^ is positioned close to Asn127 and His319. Some other receptor residues present in the vicinity of TAPP are shown in Figure 2B. Furthermore, an intramolecular hydrogen bond between C=O of d-Ala^2^ and N-H of Phe^4^ was predicted by docking.

**Figure 3:**
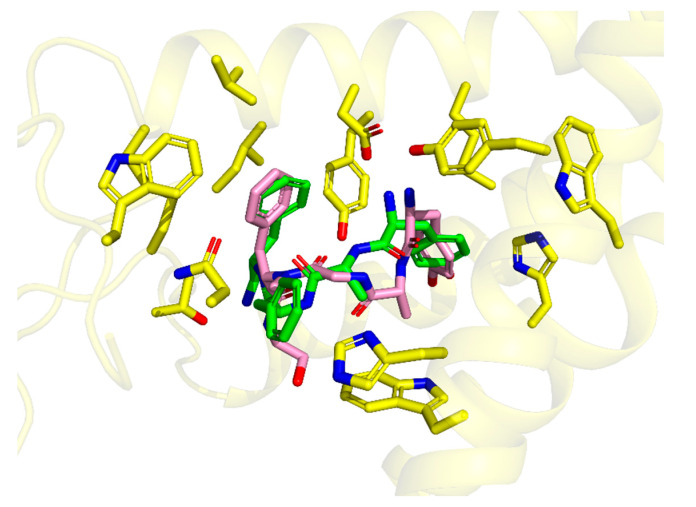
Comparison of the binding modes of DAMGO (from 6DDF PDB structure [31], pink sticks) and TAPP (from docking, green sticks). The picture is oriented in the same projection as Figure 2. Only several side-chains of the receptor binding site (yellow sticks) are shown.

This binding mode is highly similar (Figure 3) to the one experimentally found for DAMGO in 6DDE and 6DDF structures [31]. In particular, both modes share (i) the canonical ionic interaction with Asp147, (ii) similar positioning of Tyr^1^ ring, and (iii) location of Phe^4^ ring in the same hydrophobic subsite. On the other hand, the d-Ala^2^ in TAPP is shifted more towards TM7 and to the binding site outlet when compared to DAMGO. The placement of TAPP’s Phe^4^ aromatic ring is also similar to the location of aromatic rings in small molecular ligands like BU72 (as found in crystallography [33]) or fentanyl (as found by molecular modelling [34,35]).

**Figure 4:**
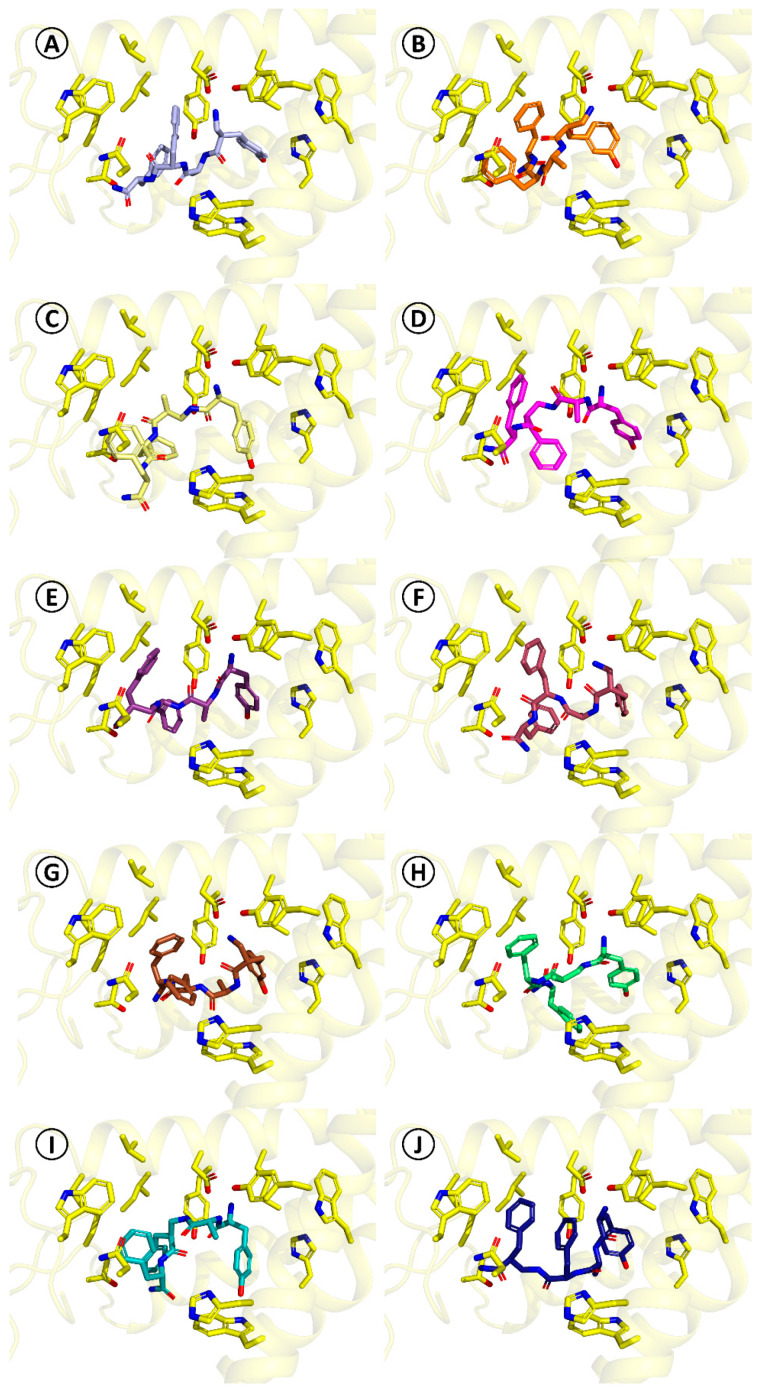
Binding modes of compounds **8**–**17** as predicted by docking. The pictures are oriented in the same projection as Figure 2. Only several side-chains of the receptor binding site (yellow sticks) are shown. (**A**) [(*R*)-β^2^hTyr^1^]-TAPP*, **8**, (**B**) [(*R*)-β^2^h-*m*-Tyr^1^]-TAPP*, **9**, (**C**) [(*S*)-β^2^hAla^2^]-TAPP, **10**, (**D**) [(*R*)-β^2^hPhe^3^]-TAPP, **11**, (**E**) [(*R*)-β^2^hPhe^4^]-TAPP, **12**, (**F**) [(*S*)-β^2^hTyr^1^]-TAPP*, **13**, (**G**) [(*S*)-β^2^h-*m*-Tyr^1^]-TAPP*, **14**, (**H**) [(*R*)-β^2^hAla^2^]-TAPP, **15**, (**I**) [(*S*)-β^2^hPhe^3^]-TAPP, **16**, (**J**) [(*S*)-β^2^hPhe^4^]-TAPP, **17**.

For the herein reported α/β-peptides (**8–17**), the docking predicts that they do not necessarily adopt the binding mode found for TAPP. The obtained binding poses are presented in Figure 4 and they are summarized in detail in Table SM-MOD-1 in Supplementary Materials. A feature common for all studied derivatives is the presence of the canonical ionic interaction with Asp147. On the other hand, the side chain of Tyr^1^ may be displaced in comparison to TAPP or DAMGO, participating in different sets of interactions. Furthermore, in some cases, docking predicts that the peptide bond elements may form hydrogen bonding to Tyr148. The aromatic ring in position three can be located either closer to TM7 (as in TAPP) or closer to ECL2 and TM2. With regard to position four, for majority of the derivatives, the aromatic ring is situated in the hydrophobic pocket formed by TM3, ECL1, and ECL2, as in the case of TAPP and DAMGO. However, the position of the ring can be more or less displaced in comparison to these ligands and utterly different positions are found as well. Furthermore, the interactions of the C-terminal amide are also predicted to vary depending on the derivative.

**Figure 5:**
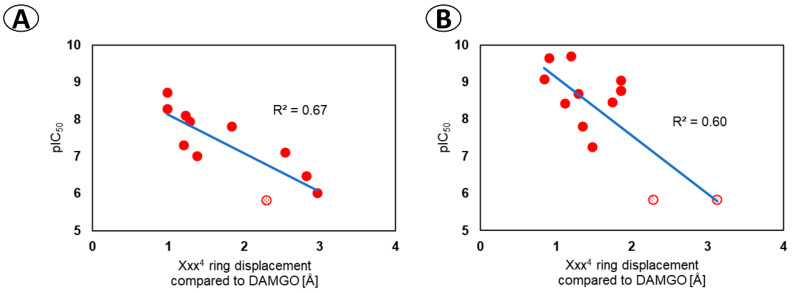
Correlation between the displacement of the ring of the fourth residue predicted by docking and the experimental µOR affinity. The relationship for (**A**) the α/β-peptides reported in this work, (**B**) derivatives reported in Reference [9]. The ring displacement is expressed as the RMSD of Xxx^4^ ring in the considered derivative (as predicted by docking) compared to *N*-Me-Phe^4^ ring in DAMGO (6DDF structure [31]). The points marked with dotted texture are arbitrarily chosen 5.82 for pIC_50_ being less than 6.

Among this diversity of binding poses, it is hard to establish a coherent relationship between the modification in the peptide structure and the change in the peptide-receptor interactions. The considered expansions of the backbone affect, in most cases, more than one interaction site. Unfortunately, in quantitative terms, the scoring function used for docking is not able to provide a predictive tool for foreseeing the effects of such modest structural changes since no correlation was found between the scoring value and the experimental affinity (Figure SM-MOD-1 and SM-MOD-2 in Supplementary Materials). The analysis of the binding poses in qualitative terms allowed however for detection of an interesting relationship between the position of the Xxx^4^ side chain (according to docking) and the affinity. The derivatives with the worst binding results are predicted to have the aromatic ring of the fourth residue significantly displaced when compared to DAMGO or TAPP. This can be quantitatively expressed in the form of a correlation equation (Figure 5A) where the independent variable standing for the ring displacement is the root mean square deviation (RMSD) of Xxx^4^ atoms’ positions of a considered derivative when compared to DAMGO in the 6DDF structure [31]. It turns out that this computational value correlates with the experimental affinity with a coefficient of determination (R^2^) of 0.67, which can be considered a fairly good explanatory power for such a model.

We were then curious to see whether this relationship (of Xxx^4^ position and affinity) might have some general validity. In order to check it, we have docked another 12 H-Tyr-Xxx-Yyy-Phe-NH_2_ derivatives (Xxx = Ala, d-Ala, β-Ala, *N*-MeAla, *N*-MeGly, Yyy = Phe or Trp) that were experimentally tested for µOR affinity by Perlikowska et al. [9]. For these compounds, the Xxx^4^ ring RMSD correlated against affinity with R^2^ = 0.60 (Figure 5B). In light of these results, it seems reasonable to conclude that, for closer or more remote TAPP derivatives, a critical requirement for high µOR affinity is the ability to place the Xxx^4^ ring in the manner similar to that of DAMGO (that is, in the hydrophobic subpocket formed by residues of TM3, ECL1, and ECL2).

**Figure 6:**
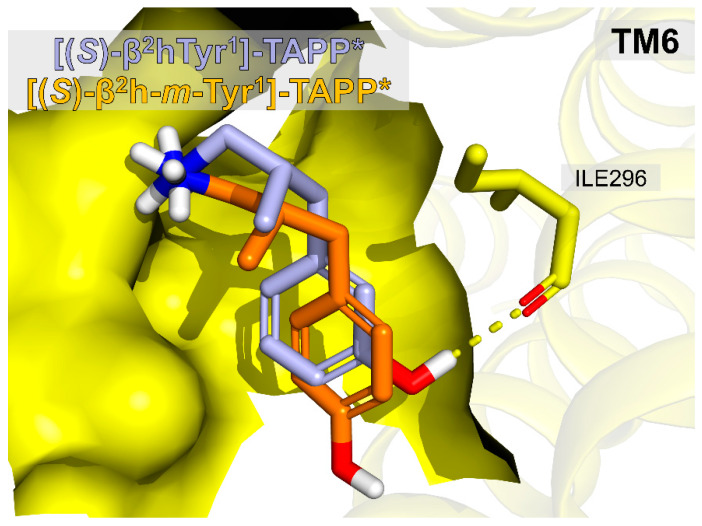
Comparison of binding poses of compounds [(*S*)-β^2^hTyr^1^]-TAPP*, **13** (orange) and [(*S*)-β^2^h-*m*-Tyr^1^]-TAPP*, **14** (light blue). Only the first residues of the peptides are shown. The receptor is partially represented as a surface representing the binding site. Only Ile296 side-chain (yellow sticks) is explicitly shown.

A pair of derivatives that deserves a closer structural look are [(*S*)-β^2^hTyr^1^]-TAPP* (**13**, IC_50_ = 338.8 ± 5.1 nM) and [(*S*)-β^2^h-*m*-Tyr^1^]-TAPP* (**14**, IC_50_ = 11.2 ± 6.3 nM). In this case, the minute difference in positioning of the phenol group (*para*- vs. *meta*-) is associated with over a thirty-fold difference in affinity. Our modelling seems to provide explanation for this effect. Figure 6 gives a comparison of the binding poses of **13** and **14**. β^2^-*Homo*logation in the first position backbone accompanied with the inversion of the side chain spatial positioning causes the peptide **13** to adopt the binding pose of an apparently higher energetics than the parent. This can be associated with an additional rotor being penalized for entropy and no novel interactions being created. On the other hand, if the phenol group is shifted to the meta-position of the aromatic ring ([(*S*)-β^2^h-*m*-Tyr^1^]-TAPP*, **14**), it allows for forming an H-bond to backbone carbonyl of Ile296 and this restores the affinity.

### 2.4. Stability Against Proteolysis in Plasma

**Figure 7:**
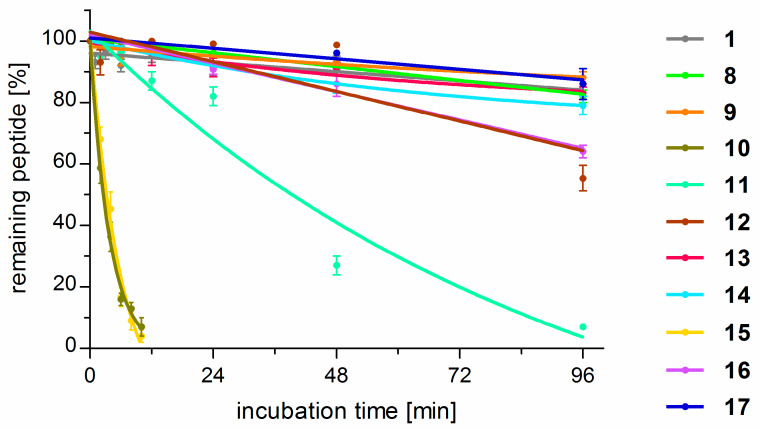
Results of stability determinations for compounds **1** and **8**–**17** expressed as percent of the initial peptide concentration remaining in the test solution at a certain time-point. Each point is an average result ± standard deviation calculated from three independent experiments.

The parent peptide (**1**) and the analogues (**8**–**17**) were tested as to stability against proteolysis in human plasma by a HPLC/MS method [19]. The results are graphically presented in Figure 7 and the representative HPLC chromatograms are given in Figures SM-STAB-1 to SM-STAB-22 in Supplementary Materials. TAPP turned out to be resistant to proteolysis. After 96 h, more than 90% of the initial peptide concentration (C_96h_ > 90%) remained in the test sample. This is in marked contrast to endomorphin-2 (Tyr-Pro-Phe-Phe-NH_2_) that, in the very same conditions, is rapidly degraded with half-life time (T_1/2_) of only 30 min. For TAPP, the first proteolytic cleavage occurs at the C-terminal amide (deamidation).

Several of the studied analogues were much less stable when compared to the parent compound. In particular, replacement of d-Ala (**1**) for both (*S*)-β^2^hAla (**10**) or (*R*)-β^2^hAla (**15**) in position 2 gave compounds prone to proteolysis with T_1/2_ of about 4.5 h. Derivatives with β^2^hPhe in the third position were also less stable than the parent, but different rates of cleavage were observed for the stereoisomers. [(*R*)-β^2^hPhe^3^]-TAPP (**11**) was found to have half-life time of about 40 h, while, in the case of [(*S*)-β^2^hPhe^3^]-TAPP (**16**), more than 65% of the initial peptide concentration remained in the test solution after 96 h. When modifications in the fourth position are considered, again the isomer with (*R*)-β^2^hPhe (**12**) was less stable (C_96h_ = 55%) than the peptide with reverse configuration in this position (**17**, C_96h_ ~ 90%). The latter was equally resistant to proteolysis as the parent. All derivatives with β^2^-*Homo*-amino acids in positions 2, 3, and 4 were cleaved at the peptide bond between the Tyr^1^ and the second residue (the site of the first proteolytic cleavage).

On the contrary, for the analogues modified in the first position, the first cleavage occurred at the C-terminal amide (deamidation) in the case of the parent compound. All they (**8**, **9**, **13**, **14**) exhibited stability similar to TAPPs.

## 3. Materials and Methods 

### 3.1. Chemistry

All materials (solvents and reagents) were purchased from commercial suppliers and used without further purification. The NMR spectra were recorded on a Varian Unity Plus 200 spectrometer operating at 200 MHz for ^1^H-NMR and 50 MHz for ^13^C-NMR. The spectra were measured in CDCl_3_ or CD_3_OD or acetone d_6_ and are given as δ values (in ppm) relative to TMS. Melting points were determined on a Melting Point Meter KSP1D (A. Krüss Optronic, Hamburg, Germany). TLC analyses were performed on silica gel plates (Merck Kiesegel GF254, Merck, Darmstadt, Germany) and visualized using UV light or iodine vapour or ninhydrin test. Column chromatography was carried out (at atmospheric pressure) using Silica Gel 60 (230–400 mesh, Merck, Darmstadt, Germany) using appropriate eluents. The crude final peptides were purified using reversed-phase high performance liquid chromatography (RP-HPLC) on a preparative C-12 column (Phenomenex, Jupiter 4u Proteo 90A, AXIA 250 × 21.20 mm) using 0.1% trifluoroacetic acid (TFA) in water/acetonitrile as a solvent system with UV detection (214 nm). The peptide purity was estimated by analytical HPLC using a C-12 column (Jupiter 4u Proteo 90A, 250 × 4.6 mm) and the same solvent system and UV detection as above, and then confirmed in a second solvent system 0.1% TFA in water/methanol. High resolution mass spectra (also low resolution) were acquired on the Shimadzu LCMS-IT TOF mass spectrometer with electrospray ionization (ESI).

### 3.2. Synthesis of β^2^-Homo-Amino Acids

#### 3.2.1. Methyl α-methyl-cyanoacetate (**3a**)

To a stirred solution of methyl cyanoacetate **2** (30 mmol, 1 equiv) and anhydrous potassium carbonate (30 mmol, 1 equiv) in dimethylformamide, methyl iodide (30 mmol, 1 equiv) was added. Then the reaction mixture was stirred overnight. Next, the reaction mixture was diluted with water and extracted with diethyl ether (4 ×). The combined organic layers successively washed with water (2 ×), brine solution (3 ×), dried over anhydrous Na_2_SO_4_, and evaporated under vacuum. The crude compound was purified by column chromatography on silica gel (hexane/ethyl acetate). Pure product **3a** was obtained as colourless, low-density oil (1.42 g, 42%).

^1^H NMR (200 MHz, CDCl_3_) δ: 3.82 (s, 3H), 3.42 (q, *J* = 7.6 Hz, 1H), 1.37 (d, *J* = 7.6 Hz, 3H). ^13^C NMR (50 MHz, CDCl_3_) δ: 165.9, 117.35, 53.24, 30.57, 15.30.

#### 3.2.2. General Procedure (GP1) of Methyl α-cyano-cinnamates Synthesis via Knoevenagel Condensation (**3b**–**d**)

The benzaldehyde (30 mmol, 1 equiv) or 3- or 4-hydroxybenzaldehyde, methyl cyanoacetate **2** (30 mmol, 1 equiv) and piperidine (0.2 mmol, 0.007 equiv) were heated under reflux for 8–12 h in methanol. The reaction mixture was allowed to reach room temperature. Then volatiles were evaporated. The crude compounds **3b**–**d** were purified by column chromatography (hexane/ethyl acetate) or crystallization from methanol.

##### Methyl α-cyanocinnamate (**3b**)

The desired product was isolated by column chromatography (hexane/ethyl acetate) or crystallization (methanol) as white solid (4.98 g, 89%), mp = 90–91 °C [lit. 89–90 °C] [36].

^1^H NMR (200 MHz, CDCl_3_) δ: 8.27 (s, 1H), 8.06–7.88 (m, 2H), 7.60–7.42 (m, 3H), 3.94 (s, 3H). ^13^C NMR (50 MHz, CDCl_3_) δ: 162.94, 155.28, 133.38, 131.33, 131.06, 129.25, 115.41, 102.47, 53.36.

##### Methyl α-cyano-4-hydroxycinnamate (**3c**)

The desired product was isolated by crystallization (twice from methanol) as a pale yellow solid (4.63 g, 76%), mp = 210–212 °C [lit. 208–210 °C] [37]

^1^H NMR (200 MHz, CD_3_OD) δ: 8.21 (s, 1H), 7.98–7.88 (m, 2H), 6.95–6.87 (m, 2H), 3.88 (s, 3H). ^13^C NMR (50 MHz, CD_3_OD) δ: 165.09, 164.46, 156.13, 135.17, 124.49, 117.30, 115.45, 98.58, 53.50.

##### Methyl α-cyano-3-hydroxycinnamate (**3d**)

The desired product was isolated by crystallization (twice from methanol) as a yellow solid (4.81 g, 79%), mp = 139–140 °C [lit. 142–143 °C] [38]

^1^H NMR (200 MHz, CD_3_OD) δ: 8.21 (s, 1H), 7.47–7.34 (m, 3H), 7.05–6.99 (m, 1H), 3.90 (s, 3H). ^13^C NMR (50 MHz, CD_3_OD) δ: 164.41, 159.42, 156.61, 134.21, 131.48, 124.04, 121.93, 117.90, 116.48, 103.57, 53.91.

#### 3.2.3. (*R*,*S*)-*N*-Boc-β^2^-*Homo*-Alanine Methyl Ester (**4a**)

To a stirred solution of the methyl α-methyl-cyanoacetate **3a** (10 mmol, 1 equiv), Boc anhydride (20 mmol, 2 equiv), CoCl_2_·6H_2_O (2 mmol, 0.2 equiv) in dry methanol at 0 °C, sodium borohydride (70 mmol, 7 equiv) were added gradually over 30 min. The reaction mixture was then stirred overnight at room temperature. Then, triethylamine (10 mmol, 1 equiv) was added and stirring was continued for 30 min. Next, the solvent was evaporated and the residue was diluted with ethyl acetate and extracted with saturated aqueous NaHCO_3_ (3 ×). The organic layer was washed with brine solution and dried over anhydrous Na_2_SO_4_, and then evaporated under vacuum. The product (Boc-β^2^hAla-OMe, **4a**) was isolated by column chromatography on silica gel (hexane/ethyl acetate) as solidifying, colorless oil (1.30 g, 60%).

^1^H NMR (200 MHz, CDCl_3_) δ: 4.86 (br s, 1H), 3.71 (s, 3H), 3.54–3.12 (dm, 2H), 2.54–2.36 (m, 1H), 1.43 (s, 9H), 1.12 (d, *J* = 7 Hz, 3H). ^13^C NMR (50 MHz, CDCl_3_) δ: 175.07, 155.77, 79.24, 52.15, 43.18, 39.58, 28.31, 15.91. LR–MS *m/z*: 140 [M–Boc + Na]^+^, 240 [M + Na]^+^.

#### 3.2.4. General Procedure (GP2) of Aromatic β^2^-*Homo*-Amino Acid Synthesis, Boc-β^2^hXaa-OMe (**4b**–**d**)

To a stirred solution of the suitable methyl α-cyano-cinnamate derivative **3b**–**d** (10 mmol, 1 equiv), Boc anhydride (40 mmol, 4 equiv), CoCl_2_·6H_2_O (4 mmol, 0.4 equiv) in dry methanol at 0 °C, and sodium borohydride (140 mmol, 14 equiv) were added gradually over 30 min. The reaction mixture was then stirred overnight. Then, triethylamine (20 mmol, 2 equiv) was added and stirring was continued for 30 min. Next, the solvent was evaporated and the residue was diluted with ethyl acetate and extracted with saturated aqueous NaHCO_3_ (3 ×). The organic layer was washed with brine solution, dried over anhydrous Na_2_SO_4_, and then evaporated under vacuum. The crude products **4b**–**d** were isolated by column chromatography on silica gel (hexane or chloroform/ethyl acetate).

##### (*R*,*S*)-*N*-Boc-β^2^-*Homo*-phenylalanine methyl ester (**4b**)

The desired product (Boc-β^2^hPhe-OMe, **4b**) was purified by column chromatography (hexane/ethyl acetate) as a solidifying oil (1.61 g, 55%).

^1^H NMR (200 MHz, CDCl_3_) δ: 7.33–7.13 (m, 5H), 4.86 (br s, 1H,), 3.64 (s, 3H), 3.48–3.16 (m, 2H), 3.10–2.74 (m, 3H), 1.42 (s, 9H). ^13^C NMR (50 MHz, CDCl_3_) δ: 174.61, 155.73, 138.22, 128.80, 128.49, 126.56, 79.38, 51.77, 47.35, 41.48, 35.85, 28.30. LR-MS *m/z*: 316.2 [M + Na]^+^.

##### (*R*,*S*)-*N*-Boc-β^2^-*Homo*-*O*-tert-butyloxycarbonyl-tyrosine methyl ester (**4c**)

The desired product (Boc-β^2^hTyr(Boc)-OMe, **4c**) was purified by column chromatography (chloroform/ethyl acetate) as a solidifying oil (2.01 g, 49%).

^1^H NMR (200 MHz, acetone d_6_) δ: 7.32–7.16 (m, 2H), 7.16–6.96 (m, 2H), 6.14 (br s, 1H), 3.57 (s, 3H), 3.38–3.20 (m, 2H), 3.0–2.76 (m, 3H), 1.52 (s, 9H), 1.40 (s, 9H), ^13^C NMR (50 MHz, acetone d_6_) δ: 174.59, 152.66, 150.81, 137.62, 130.71, 122.07, 83.50, 79.38, 51.89, 48.81, 42.84, 35.61, 28.64, 27.83. LR-MS *m/z*: 432.2 [M + Na]^+^.

##### (*R,S*)-*N*-Boc-β^2^-*Homo*-*O*-*tert*-butyloxycarbonyl-*meta*-tyrosine methyl ester (**4d**)

The desired product (Boc-β^2^h-*m*-Tyr(Boc)-OMe, **4d**) was purified by column chromatography (chloroform/ethyl acetate) as a solidifying oil (1.92 g, 47%).

^1^H NMR (200 MHz, acetone d_6_) δ: 7.38–7.20 (m, 1H), 7.16–6.90 (m, 3H), 6.14 (br s, 1H), 3.57 (s, 3H), 3.38–3.24 (m, 2H), 3.0–2.78 (m, 3H), 1.52 (s, 9H), 1.40 (s, 9H). ^13^C NMR (50 MHz, acetone d_6_) δ: 176.59, 156.37, 150.81, 143.25, 142.08, 130.61), 127.65, 121.93, 118.07, 83.63, 79.10, 51.70, 48.81, 42.84, 35.35, 28.31, 27.80. LR-MS *m/z*: 432.2 [M + Na]^+^, 841.4 [2M + Na]^+^.

### 3.3. Separation of Racemic β^2^-Homo-Amino Acids (**7a**–**b**)

Boc-protected β^2^-*Homo*-amino acids **5a**–**b** (also **5c**–**d**) were obtained according to the GP4. To a stirred solution of **5a**–**b** (5 mmol, 1 equiv) in DMF (10 mL), TBTU (5 mmol, 1 equiv), HOBt (5 mmol, 1 equiv), and DIPEA (10 mmol, 2 equiv) were added. After stirring the mixture at 0 °C for 10 min, the (S)-(−)-α-methylbenzylamine (5 mmol, 1 equiv) was added. The reaction mixture was then stirred overnight (allowing to reach to room temperature). Next, the mixture was diluted with 5% NaHCO_3_ and washed several times with ethyl acetate. The combined organic layers successively washed with 5% NaHCO_3_ (2 ×), 1M KHSO_4_ (3 ×), brine solution (3 ×), and dried over anhydrous Na_2_SO_4_ and evaporated under vacuum. The resulting diastereoisomeric amide pairs (**6a**–**b**) were separated by column chromatography (supplemented by an additional re-chromatography of partially separated fractions) using as eluent CHCl_3_/AcOEt for **6a** or hexane/AcOEt for **6b**. The individual amide diastereoisomers were heated under reflux in 6M HCl for 12 to 18 h. The reaction progress was monitored by TLC. After the reaction, the mixture was concentrated, and respective (*R*)- or (*S*)-**7a**–**b** enantiomers were isolated using cation exchange ion chromatography (Dowex 50W X12). A 0.2 M ammonia solution was used as an eluent. After evaporation, white solids of individual isomers were obtained and dried over NaOH.

#### 3.3.1. (*R*)-(−)-β^2^-*Homo*-alanine, (*R*)-(−)-**7a**

The desired product (*R*)-(−)-β^2^hAla was obtained as white solid, mp 186–187 °C [lit. 185–187 °C] [39]; [α]^20^_D_ − 10.7 (c 1.0, 1M HCl) [lit. [α]^29^_D_ − 11.8 (c 1.0, 1M HCl)] [39].

^1^H NMR (200 MHz, CD_3_OD) δ: 3.26–3.10 (dd, *J* = 8.6 Hz, *J* =12.8 Hz, 1H), 3.10–2.96 (dd, *J* = 4.8 Hz, *J* = 12.8 Hz, 1H), 2.96–2.76 (m, 1H), 1.31 (d, *J* = 7.0 Hz, 3H). ^13^C NMR (50 MHz, CD_3_OD) δ: 176.71, 42.68, 38.18, 15.33. LR–MS *m/z*: 104.1 [M + H]^+^, 207.2 [2M + H]^+^.

#### 3.3.2. (*S*)-(+)-β^2^-*Homo*-alanine, (*S*)-(+)-**7a**

The desired product (*S*)-(+)-β^2^hAla was obtained as a white solid, mp 187–188 °C [lit. 184–188 °C] [39]; [α]^20^_D_ + 10.2 (c 0.96, 1M HCl) [lit. [α]^29^_D_ + 11.6 (c 1.0, 1M HCl)] [39].

^1^H NMR (200 MHz, CD_3_OD) δ: 3.30–3.12 (dd, *J* = 8.4 Hz, *J* = 13 Hz, 1H), 3.10–2.92 (dd, *J* = 5 Hz, *J* = 13 Hz, 1H), 2.92–2.80 (m, 1H), 1.27 (d, *J* = 7.0 Hz, 3H). ^13^C NMR (50 MHz, CD_3_OD) δ: 176.76, 42.88, 38.98, 15.43. LR-MS *m/z*: 104.1 [M + H]^+^, 207.2 [2M + H]^+^.

#### 3.3.3. (*R*)-(+)-β^2^-*Homo*-phenylalanine, (*R*)-(+)-**7b**

The desired product (*R*)-(+)-β^2^hPhe was obtained as a white solid, mp 210–212 °C [lit. 225–226 °C] [39]; [α]^20^_D_ + 13.8 (c 1.083, 1M HCl) [lit. [α]^29^_D_ + 11.3 (c 1.0, 1M HCl)] [39].

^1^H NMR (200 MHz, CD_3_OD) δ: 7.35–7.28 (m, 2H), 7.20–6.87 (m, 3H), 3.12–2.90 (m, 3H), 2.80–2.54 (m, 2H). ^13^C NMR (50 MHz, CD_3_OD) δ: 176.61, 138.22, 129.80, 128.49, 126.56, 46.35, 40.48, 36.85. LR-MS *m/z*: 180.1 [M + H]^+^, 359.2 [2M + H]^+^.

#### 3.3.4. (*S*)-(–)-β^2^-*Homo*-phenylalanine, (*S*)-(−)-**7b**

The desired product (*S*)-(−)-β^2^hPhe was obtained as a white solid, mp 212–214 °C [lit. 224–225 °C] [39]; [α]^20^_D_ − 9.8 (c 0.835, 1M HCl) [lit. [α]^29^_D_ − 11.0 (c 1.0, 1M HCl)] [39].

^1^H NMR (200 MHz, CD_3_OD) δ: 7.37–7.28 (m, 2H), 7.30–6.95 (m, 3H), 3.14–2.90 (m, 3H), 2.80–2.56 (m, 2H). ^13^C NMR (50 MHz, CD_3_OD) δ: 177.61, 139.20, 128.80, 128.59, 127.56, 45.35, 41.44, 36.67. LR-MS *m/z*: 180.1 [M + H]^+^, 359.2 [2M + H]^+^.

### 3.4. Synthesis of the Peptides

All planned tetrapeptides (**1**, **8**–**17**) were obtained according to the following steps of the synthesis: (I) preparation of Boc-protected N-terminal dipeptide methyl ester by the GP3, (II) preparation of Boc-protected C-terminal dipeptide methyl ester using the *GP3*, (III) hydrolysis of the methyl ester of the product of step (I) according to GP4, (IV) Boc-deprotection of Boc-protected C-terminal dipeptide methyl ester (step II) using GP5, (V) coupling of Boc-protected N-terminal dipeptide to C-terminal dipeptide methyl ester according to GP3, (VI) aminolysis of Boc-protected tetrapeptide methyl ester by the GP6. (VII) purification by RP-HPLC, confirmation of purity (≥ 96%) by analytical RP-HPLC and confirmation of molecular weight by mass spectrometry (3.1. Chemistry). HR-MS: for TAPP (**1**) 546.2715 (calcd for C_30_H_35_N_5_O_5_, 546.2711), for [(*R*)-β^2^hTyr^1^]-TAPP* (**8**) 560.2885, for [(*R*)-β^2^h-*m*-Tyr^1^]-TAPP* (**9**) 560.2885, for [(*S*)-β^2^hAla^2^]-TAPP (**10**) 560.2893, for [(*R*)-β^2^hPhe^3^]-TAPP (**11**) 560.2879, for [(*R*)-β^2^hPhe^4^]-TAPP (**12**) 560.2889, for [(*S)*-β^2^hTyr^1^]-TAPP* (**13**) 560.2874, for [(*S*)-β^2^h-*m*-Tyr^1^]-TAPP* (**14**) 560.2879, for [(*R*)-β^2^hAla^2^]-TAPP (**15**) 560.2880, for [(*S*)-β^2^hPhe^3^]-TAPP (**16**) 560.2879, for [(*S*)-β^2^hPhe^4^]-TAPP (**17**) 560.2882 (for **8**–**17** calcd for C_31_H_37_N_5_O_5_, 560.2867). The analytical data for compounds **1** and **8**–**17** are summarized in Supplementary Materials (Table SM-SYN-1).

#### 3.4.1. General Procedure (GP3) for Synthesis of Protected Dipeptides and Tetrapeptides 

To stirred solution of Boc-amino acid or Boc-dipeptide acid (10 mmol) in DMF (10mL), TBTU (10 mmol, 1 equiv), HOBt (10 mmol, 1 equiv), and DIPEA (30 mmol, 3 equiv) were added. After stirring the mixture at 0 °C for 10 min, the amine component or dipeptide methyl ester (10 mmol, 1 equiv) was added. The reaction mixture was then stirred overnight (allowing to reach the room temperature). Next, the mixture was diluted with 5% NaHCO_3_ and washed several times with ethyl acetate. The combined organic layers successively washed with 5% NaHCO_3_ (2 ×), 1M KHSO_4_ (3 ×), brine solution (3 ×), and dried over anhydrous Na_2_SO_4_ and evaporated under vacuum. The crude peptides were used for the next step without further purification.

#### 3.4.2. General Procedure (GP4) for Methyl ester Removal

To a stirred solution of Boc-protected dipeptide methyl ester (10 mmol, 1 equiv) in MeOH, LiOH·H_2_O (50 mmol, 5 equiv) was added. The reaction mixture was stirred at 0 °C for 4–8 h with TLC-monitoring of the reaction progress. Next, the solvent was evaporated under reduced pressure. The residue was diluted with water and washed with diethyl ether (2 ×), acidified up to pH = 3 with 10% citric acid (aq.), and extracted with ethyl acetate (3 ×). The organic layer was dried over anhydrous MgSO_4_, and concentrated under vacuum and used for the next step without further purification.

#### 3.4.3. General Procedure (GP5) for Boc-Deprotection

Boc-protected dipeptide methyl ester (10 mmol, 1 equiv) was deprotected by 1N HCl(g) in ethyl acetate (8–10 mL). The Boc-tetrapeptide amide was deprotected by 1N HCl(g) in acetic acid (8–10 mL) at room temperature for 2–3 h (TLC-monitoring). Then, the excess of cold ethyl ether was added to the reaction mixture. Precipitated product was filtered off, washed with diethyl ether, and used for the next step without further purification.

#### 3.4.4. General Procedure (GP6) for Aminolysis of Methyl ester 

The Boc–protected tetrapeptide methyl ester (20 mmol, 1 equiv) was dissolved in ammonia solution in methanol (7*N*, 10 mL) and stirred for 3 to 10 days. The progress of the reaction was monitored by HPLC. The solution was evaporated under reduced pressure and the crude product was used for the next step without further purification.

### 3.5. Binding Affinity Determinations

The binding affinity of compounds **1** and **8**–**17** for μOR and δOR was determined in competitive radioligand binding assays following the previously described method [19,40]. Membrane fractions of rat brain *Homo*genate were incubated at 25 °C for 60 mins in the presence of radioligands (0.5 nM) specific for each receptor (μOR: [^3^H]DAMGO and δOR: [^3^H]DELT II, obtained as a generous gift from Prof. Géza Tóth [41]) and the increasing concentrations of the tested compounds (10^−11^ up to 10^−5^ M, each concentration in duplicate). For measuring non-specific binding, 10 μM naloxone was used as the competitor. The assay buffer contained 50mM Tris-HCl (pH 7.4), bovine serum albumin (0.1 mg/mL), bacitracin (30 μg/mL), bestatin (30 μM), captopril (10 μM), and phenylmethylsulfonyl fluoride (0.1 mM) in the total reaction volume of 1 mL. After the incubation, the binding reactions were terminated by rapid filtration with M-24 Cell Harvester (Brandel/USA) through GF/B Whatman glass fibre strips. The filters were pre-soaked with 0.5% PEI before harvesting in order to minimize non-specific binding. Filter discs were placed separately in 24-well plates and immersed with the Optiphase Supermix scintillation cocktail (Perkin Elmer, Waltham, MA, USA). Radioactivity was measured using a MicroBeta LS Trilux scintillation counter (PerkinElmer, Waltham, MA, USA). The experiments were repeated three times in duplicate and the results are presented as mean half-maximal inhibitory concentrations (IC_50_) with standard errors of the mean.

### 3.6. Molecular Modelling

The compounds **1** and **8**–**17** were docked into activated µOR structure (PDB accession code: 6DDF [31]) using AutoDock 4.2.6 [32]. The studied derivatives were sketched in Biovia Discovery Studio Visualizer [42] and minimized with a Dreiding-like force field. Furthermore, they and the protein structure were processed in AutoDock Tools 4 [32]. Protonation states were set as expected at physiological pH. The G-protein was removed from the receptor structure. The ligands were considered flexible (except for amide bonds of the backbone) and the receptor was set as rigid. The docking box was centered around the position of DAMGO in the 6DDF structure [31] and its size was extended to cover the binding pocket and the extracellular part of the binding pocket entry. Grids were calculated with AutoGrid and the docking was performed using Lamarckian Genetic Algorithm with pseudo-Solis and Wets local searches (300 runs). A genetic algorithm was set to work with a population of 3000 individuals with a maximum number of generations being 37,000, mutation rate of 0.02, crossover rate of 0.8, and one individual surviving to the next generation. The probability of local search on the individual was set to 0.1. The results of each run were clustered and representative poses from several top-scored clusters of each ligand were visually inspected. A criterion for selecting the binding poses was their conformity to known literature data on ligand-µOR interactions, i.e., presence of the interaction of protonated amine in position 1 with Asp147. Molecular graphics were prepared in PyMOL [43].

In order to validate the relationship presented in Figure 5, a further 12 derivatives [9] were docked to the µOR structure using the very same procedure as described above.

### 3.7. Stability Determinations

The determination of plasma stability was performed according to a previously described protocol with minor modifications [19]. Human plasma for testing was obtained from a healthy donor. Compounds **1** and **8**–**17** (0.7 μmol) were dissolved in water to obtain 1 mL of stock solution. Portions of the plasma (100 μL) were dispensed into Eppendorf tubes and equilibrated at 37 ± 1 °C for 5 min, before adding 100 μL of the stock solution of each tested peptide. Samples of the test solution were taken and analyzed at several time points (0, 1, 2, 3, 4, 5, 6, 12, 24, 48, and 96 h of the experiment).

In order to precipitate the plasma proteins, 400 μL ethanol (98%) was added to the samples, whereafter shaking (1 min) and cooling in 4 °C (5 min) followed. Subsequently, the samples were centrifuged for 10 min at 2000 g. The supernatant (20 μL) was immediately analyzed by HPLC/MS using the LCMS-2010EV Shimadzu apparatus with a Phenomenex Jupiter 4u Proteo 90A, C12 (25 cm × 2 mm × 4 μm) column. The chromatography was performed in reversed-phase system (solvents A: 0.05% FA in water, B: 0.05% FA in acetonitrile) with a non-linear gradient was used (1–31% B in 20 min followed by an increase to 97% B from 20 to 30 min) at a solvent flow of 0.5 mL/min. The activity of the plasma was confirmed by using endomorphin-2 (0.7 μM/mL) as a control sample. The results of the HPLC/MS analyses allowed for quantitative determination of the concentration changes of the tested peptides. Furthermore, it was possible to indicate the location of the first proteolytic cleavage.

## 4. Conclusions

In conclusion, we have presented in this paper the results of β^2^hAA scan of the TAPP sequence. Ten novel analogues were synthesized as well as tested for opioid binding and stability. None of the new compounds bound the µOR better than the parent, but the compounds modified at position four had equally good affinity as TAPP. On the contrary, β^2^-*Homo*logation in the second position gave derivatives with very low binding strength. If the third position was modified, the (*R*)-isomer was weaker than TAPP, but, in the case of the (*S*)-isomer, the decrease was only minor. β^2^hTyr in the first position caused affinity decreases (more pronounced with the (*S*)-isomer), but interestingly shifting the phenol group to the *meta*-position (β^2^h-*m*-Tyr) gave restoration of binding strength. It was particularly noticeable with [(*S*)-β^2^h-*m*-Tyr^1^]-TAPP whose IC_50_ value was statistically equal to that of TAPP and 30 times lower than that of [(*S*)-β^2^hTyr^1^]-TAPP.

The presented α/β-peptides and their parent, TAPP, were docked into the µOR structure. According to modelling, TAPP binds the receptor by a canonical ionic interaction with Asp147 and by anchoring the Phe^4^ ring in a hydrophobic subsite formed by residues of TM3, ECL1, and ECL2, while placing the Phe^3^ ring closer to TM7. For the novel analogues, docking does not predict a uniform binding mode, but a rather multitude of poses that vary at several subsites. No straightforward correlation can be found between the site of backbone expansion and the change in receptor-ligand interactions. There is also no correlation between the experimental binding affinity and the scoring value. Yet, it is possible to find a relationship between the experimental binding and the positioning of the Xxx^4^ aromatic ring and this relationship can be even expressed quantitatively. Furthermore, this correlation was validated to hold for some opioid tetrapeptides reported earlier by other authors.

With regard to stability in human plasma, TAPP turned out to be fairly stable (C_96h_ > 90% of the initial concentration). Surprisingly, the α/β-analogues were not necessarily so. For example, those with (*R/S*)-β^2^hAla in position 2 turned out to have half-life times of less than 5 h. Modifications in position 3 produced derivatives that were less stable than the parent. On the contrary, β^2^hAAs in the first position gave analogues equally stable to the parent.

The presented results enhance our understanding of structure-activity (stability) relationships of opioid peptides, and, in particular, of the TAPP sequence. Thus, they will be useful for further design of peptide analgesics.

## Figures and Tables

**Table 1:** Binding affinity of the studied compounds.

	Compound	IC_50_ ± S.E.M. [nM] ^1^
No.		μOR ^2^
**1**	TAPP	5.1 ± 3.5
**Backbone Expansion without Changing the Spatial Positioning of the Side-Chain**		
**8**	[(*R*)-β^2^hTyr^1^]-TAPP*	77.6 ± 2.4
**9**	[(*R*)-β^2^h-*m*-Tyr^1^]-TAPP*	48.9 ± 2.3
**10**	[(*S*)-β^2^hAla^2^]-TAPP	> 1000
**11**	[(*R*)-β^2^hPhe^3^]-TAPP	95.4 ± 2.5
**12**	[(*R*)-β^2^hPhe^4^]-TAPP	1.9 ± 2.4
**Backbone Expansion with Changing the Spatial Positioning of the Side-Chain**		
**13**	[(*S*)-β^2^hTyr^1^]-TAPP *	338.8 ± 5.1
**14**	[(*S*)-β^2^h-*m*-Tyr^1^]-TAPP*	11.2 ± 6.3
**15**	[(*R*)-β^2^hAla^2^]-TAPP	954.9 ± 2.9
**16**	[(*S*)-β^2^hPhe^3^]-TAPP	15.5 ± 2.5
**17**	[(*S*)-β^2^hPhe^4^]-TAPP	7.8 ± 4.0

^1^ IC_50_ ± S.E.M. Half-maximal inhibitory concentration ± standard error of the mean. Mean of three determinations in duplicate. ^2^ Radioligand: 0.5 nM [^3^H]DAMGO.

## Supplementary Materials

The following are available online. SM file is divided into sections that contain data pertaining to particular analysis subjects. Items in each section are independently numbered. The sections are: **SM-SYN** (analytical data for the synthesized peptides, 1 Table), **SM-MOD** (minor data coming from modelling, 1 Table, 2 Figures), **SM-STAB** (data from stability determinations, 22 Figures).
