# Supplementary material for: β2-Homo-Amino Acid Scan of µ-Selective Opioid Tetrapeptide TAPP
Source: Molecules. 2020 May 25;25(10):2461. doi: 10.3390/molecules25102461 (PMC7288052; doi:10.3390/molecules25102461)
Supplement: Supplementary file 1 [file molecules-25-02461-s001.pdf]

Supplementary Materials to:

β^2-*homo*-Amino acid scan of μ-selective opioid tetrapeptide TAPP

by: Dagmara Tymecka*, Piotr F. J. Lipiński, Piotr Kosson and Aleksandra Misicka*

Corresponding authors: dulok@chem.uw.edu.pl; (D.T.), misicka@chem.uw.edu.pl (A.M.)

SM-SYN: synthesis

Table SM-SYN-1. Analytical data of the synthesized peptides.

SM-BIN: binding affinity

Table SM-BIN-1. δOR binding affinity of the studied compounds.

SM-MOD: modelling

Table SM-MOD-1. Descriptive summary of the binding poses predicted by docking for TAPP and the derivatives.

Figure SM-MOD-1. Plot of pIC_{50} against the predicted by docking free energy of binding (mean in the cluster).

Figure SM-MOD-2. Plot of pIC_{50} against the predicted by docking free energy of binding (lowest in the cluster).

SM-STAB: stability

Figure SM-STAB-1. HPLC chromatogram of a TAPP (**1**) sample in human plasma, taken at the start of stability testing.

Figure SM-STAB-2. HPLC chromatogram of a TAPP (**1**) sample in human plasma, taken at 48 h of stability testing.

Figure SM-STAB-3. HPLC chromatogram of a [(*R*)-β^2hTyr^1]-TAPP* (**8**) sample in human plasma, taken at the start of stability testing.

Figure SM-STAB-4. HPLC chromatogram of a [(*R*)-β^2hTyr^1]-TAPP* (**8**) sample in human plasma, taken at 48 h of stability testing.

Figure SM-STAB-5. HPLC chromatogram of a [(*R*)-β^2h-*m*-Tyr^1]-TAPP* (**9**) sample in human plasma, taken at the start of stability testing.

Figure SM-STAB-6. HPLC chromatogram of a [(*R*)-β^2h-*m*-Tyr^1]-TAPP* (**9**) sample in human plasma, taken at 48 h of stability testing.

Figure SM-STAB-7. HPLC chromatogram of a [(*S*)-β^2hAla^2]-TAPP (**10**) sample in human plasma, taken at the start of stability testing.

Figure SM-STAB-8. HPLC chromatogram of a [(*S*)-β^2hAla^2]-TAPP (**10**) sample in human plasma, taken at 4 h of stability testing.

Figure SM-STAB-9. HPLC chromatogram of [(*R*)-β^2hPhe^3]-TAPP (**11**) sample in human plasma, taken at the start of stability testing.

Figure SM-STAB-10. HPLC chromatogram of [(*R*)-β^2hPhe^3]-TAPP (**11**) sample in human plasma, taken at 48h of stability testing.

Figure SM-STAB-11. HPLC chromatogram of [(*R*)-β^2hPhe^4]-TAPP (**12**) sample in human plasma, taken at the start of stability testing.

Figure SM-STAB-12. HPLC chromatogram of [(*R*)-β^2hPhe^4]-TAPP (**12**) sample in human plasma, taken at 48 h of stability testing.

Figure SM-STAB-13. HPLC chromatogram of a [(*S*)-β^2hTyr^1]-TAPP* (**13**) sample in human plasma, taken at the start of stability testing.

Figure SM-STAB-14. HPLC chromatogram of a [(*S*)-β^2hTyr^1]-TAPP* (**13**) sample in human plasma, taken at 48 h of stability testing.

Figure SM-STAB-15. HPLC chromatogram of a [(*S*)-β^2h-*m*-Tyr^1]-TAPP* (**14**) sample in human plasma, taken at the start of stability testing.

Figure SM-STAB-16. HPLC chromatogram of a [(*S*)-β^2h-*m*-Tyr^1]-TAPP* (**14**) sample in human plasma, taken at 48 h of stability testing.

Figure SM-STAB-17. HPLC chromatogram of a [(*R*)-β^2hAla^2]-TAPP (**15**) sample in human plasma, taken at the start of stability testing.

Figure SM-STAB-18. HPLC chromatogram of a [(*R*)-β^2hAla^2]-TAPP (**15**) sample in human plasma, taken at 4 h of stability testing.

Figure SM-STAB-19. HPLC chromatogram of a [(*S*)-β^2hPhe^3]-TAPP (**16**) sample in human plasma, taken at the start of stability testing.

Figure SM-STAB-20. HPLC chromatogram of a [(*S*)-β^2hPhe^3]-TAPP (**16**) sample in human plasma, taken at 48 h of stability testing.

Figure SM-STAB-21. HPLC chromatogram of a [(*S*)-β^2hPhe^4]-TAPP (**17**) sample in human plasma, taken at the start of stability testing.

Figure SM-STAB-22. HPLC chromatogram of a [(*S*)-β^2hPhe^4]-TAPP (**17**) sample in human plasma, taken at 48 h of stability testing.

Table SM-SYN-1. Analytical data of the synthesized peptides.

**No.**	**peptide**	**HPLC t_R (min)***	**HPLC t_R (min)****	**molecular formula**	**HR ESI-MS**		
					**calculated for $[M+H]^+$**	**measured for $[M+H]^+$**	**Diff. [ppm]**
**1**	H-Tyr-D-Ala-Phe-Phe-NH_2 (TAPP)	20.866	18.017	$C_{30}H_{35}N_5O_5$	546.2711	546.2715	0.73
**8**	H-(*R*)-β^2hTyr-D-Ala-Phe-Phe-NH_2 less polar	21.160	18.672	$C_{31}H_{37}N_5O_5$	560.2867	560.2885	3.21
**9**	H-(*R*)-β^2h-*m*-Tyr-D-Ala-Phe-Phe-NH_2 less polar	21.831	19.240	$C_{31}H_{37}N_5O_5$	560.2867	560.2885	3.21
**10**	H-Tyr-(*S*)-β^2hAla-Phe-Phe-NH_2	20.059	17.340	$C_{31}H_{37}N_5O_5$	560.2867	560.2893	4.64
**11**	H-Tyr-D-Ala-(*R*)-β^2hPhe-Phe-NH_2	19.234	16.234	$C_{31}H_{37}N_5O_5$	560.2867	560.2879	2.14
**12**	H-Tyr-D-Ala-Phe-(*R*)-β^2hPhe-NH_2	20.690	18.316	$C_{31}H_{37}N_5O_5$	560.2867	560.2889	3.93
**13**	H-(*S*)-β^2hTyr-D-Ala-Phe-Phe-NH_2 more polar	19.585	17.019	$C_{31}H_{37}N_5O_5$	560.2867	560.2874	1.25
**14**	H-(*S*)-β^2h-*m*-Tyr-D-Ala-Phe-Phe-NH_2 more polar	20.039	17.711	$C_{31}H_{37}N_5O_5$	560.2867	560.2879	2.14
**15**	H-Tyr-(*R*)-β^2hAla-Phe-Phe-NH_2	19.496	16.876	$C_{31}H_{37}N_5O_5$	560.2867	560.2880	2.32
**16**	H-Tyr-D-Ala-(*S*)-β^2hPhe-Phe-NH_2	21.037	19.467	$C_{31}H_{37}N_5O_5$	560.2867	560.2879	2.14
**17**	H-Tyr-D-Ala-Phe-(*S*)-β^2hPhe-NH_2	20.611	18.298	$C_{31}H_{37}N_5O_5$	560.2867	560.2882	2.68

*linear gradient was applied: 3–97% B for 40 min, phase (A): 0.1% TFA in water; phase (B): 0.1% TFA in ACN.

**linear gradient was applied: 30–97% B for 30 min, phase (A): 0.1% TFA in water; phase (B): 0.1% TFA in MeOH.

Table SM-BIN-1. δOR binding affinity of the studied compounds.

**No.**	**Compound**	**IC_{50} [nM]**[1]
		**δOR**[2]
**1**	TAPP	616
**backbone expansion without changing the spatial positioning of the side-chain**		
**8**[3]	[(*R*)-β^2hTyr[1]]-TAPP*	>10000
**9**[3]	[(*R*)-β^2h-*m*-Tyr[1]]-TAPP*	>10000
**10**	[(*S*)-β^2hAla[2]]-TAPP	>10000
**11**	[(*R*)-β^2hPhe[3]]-TAPP	794
**12**	[(*R*)-β^2hPhe[4]]-TAPP	708
**backbone expansion with changing the spatial positioning of the side-chain**		
**13**[3]	[(*S*)-β^2hTyr[1]]-TAPP*	>10000
**14**[3]	[(*S*)-β^2h-*m*-Tyr[1]]-TAPP*	>10000
**15**	[(*R*)-β^2hAla[2]]-TAPP	>10000
**16**	[(*S*)-β^2hPhe[3]]-TAPP	890
**17**	[(*S*)-β^2hPhe[4]]-TAPP	417

[1] IC_{50}, half-maximal inhibitory concentration mean of three determinations in duplicate. [2] Radioligand: 0.5 nM [^{3}H]DELT II. [3] Absolute configuration determined *per analogiam* to [(*R/S*)-β^2hPhe[1]]-TAPP (see Section 2.1 in the main text for details)

Table SM-MOD-1. Descriptive summary of the binding poses predicted by docking for TAPP and the derivatives.

No	Structure	Xxx1 interactions	H-bond with Tyr148	Position of Xxx^3 side-chain	Position of Xxx^4 side-chain	Interactions of C-terminal amide
	DAMGO in 6DDF	SA1	-	n/a	SD1	n/a
**1**	TAPP	SA1	-	SC1	SD1	SE1
**8**	[(*R*)-β^2hTyr^1]-TAPP*	SA1	-	SC2	SC4	SE2
**9**	[(*R*)-β^2h-*m*-Tyr^1]-TAPP*	SA2	-	SC1	SD1	SE1
**10**	[(*S*)-β^2hAla^2]-TAPP	SA3	+	SC4	SC3	SE2
**11**	[(*R*)-β^2hPhe^3]-TAPP	SA4	+	to solvent	SD1	SE2
**12**	[(*R*)-β^2hPhe^4]-TAPP	SA4	-	SC4	SD1	SE2
**13**	[(*S*)-β^2hTyr^1]-TAPP*	SA5	-	SD1	SC4	to solvent
**14**	[(*S*)-β^2h-*m*-Tyr^1]-TAPP*	SA6	-	SC1	SD1	SE1
**15**	[(*R*)-β^2hAla^2]-TAPP	SA6	+	SD1	SD2	SE3
**16**	[(*S*)-β^2hPhe^3]-TAPP	SA7	-	SC4	SC3	solvent
**17**	[(*S*)-β^2hPhe^4]-TAPP	SA1	-	SC5	SD1	SE2

SA1, dispersive contacts: Ile296, His297, Val300
SA2, dispersive contacts: Met151
SA3, H-bond: Ile296 (backbone); dispersive contacts: Ile296, Val300, Ile322
SA4, H-bond: Lys233 (backbone); dispersive contacts: Val300
SA5, dispersive contacts: Ile296, Val300, Val236
SA6, H-bond: Ile296 (backbone); dispersive contacts: Val236, Ile296
SA7, H-bond: Trp293 (indole); dispersive contacts: Ile 296, Val300
SC1, dispersive contacts: Asn127, Trp318, His319
SC2, intramolecular stack $NH^+\cdots Xxx^3$
SC3, dispersive contacts: Cys217
SC4, dispersive contacts: Leu219
SC5, intramolecular stack $Xxx^4\cdots Xxx^3$
SD1, dispersive contacts: Trp133, Ile144, Cys217
SD2, dispersive contacts: Val300, Trp318
SE1, H-bond: Thr218
SE2, H-bond: Asp216
SE3, H-bond: Glu229

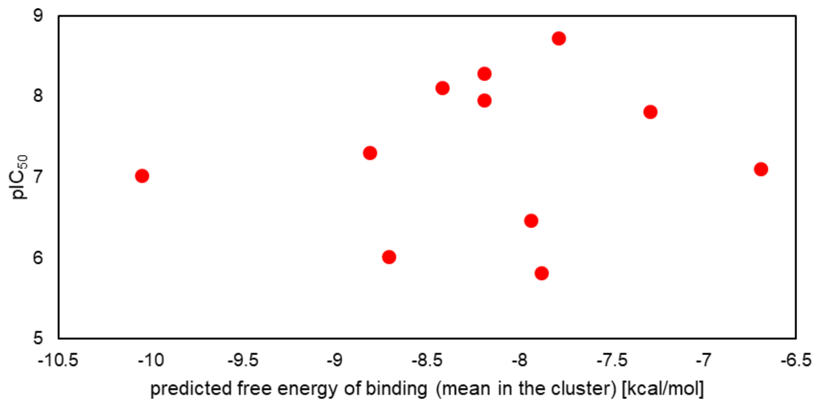

Figure SM-MOD-1. Plot of pIC_{50} against the predicted by docking free energy of binding (mean in the cluster).

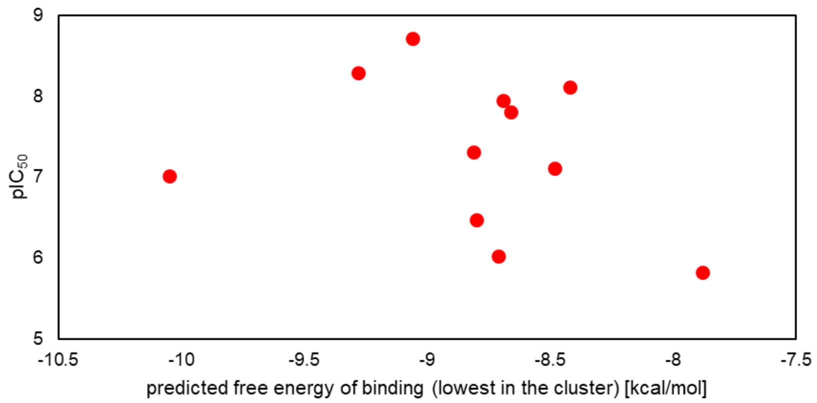

Figure SM-MOD-2. Plot of pIC_{50} against the predicted by docking free energy of binding (lowest in the cluster).

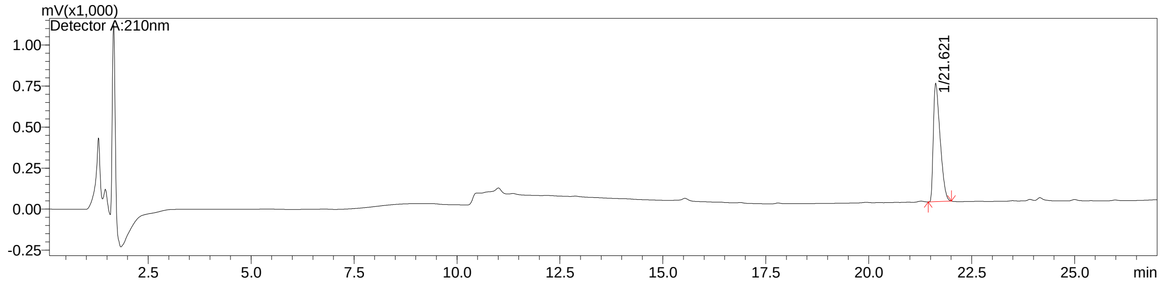

Figure SM-STAB-1. HPLC chromatogram of a TAPP (**1**) sample in human plasma, taken at the start of stability testing.

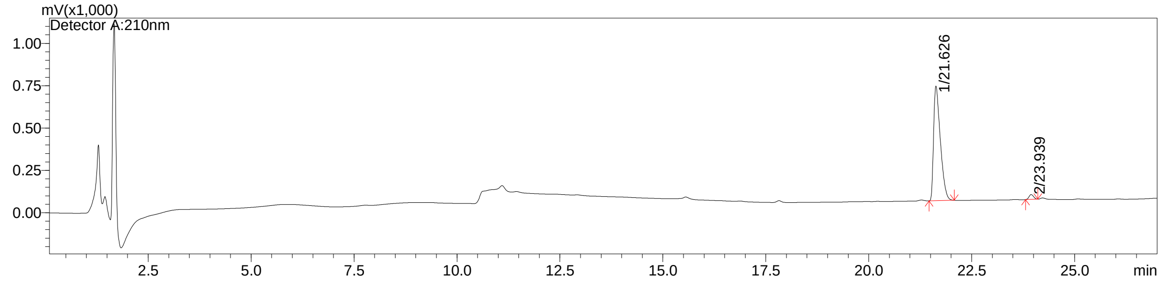

Figure SM-STAB-2. HPLC chromatogram of a TAPP (**1**) sample in human plasma, taken at 48 h of stability testing.

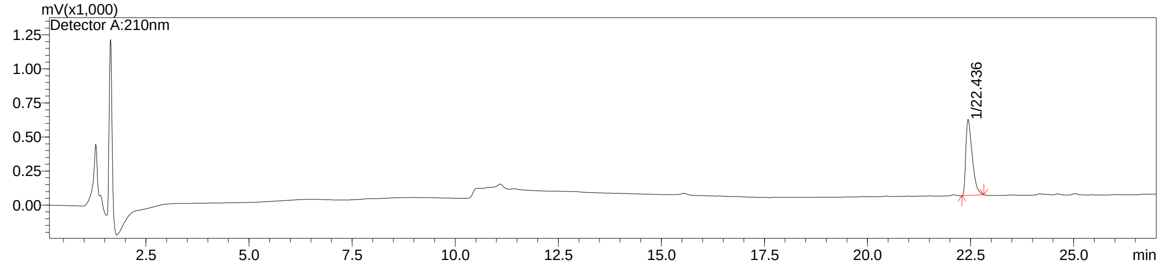

Figure SM-STAB-3. HPLC chromatogram of a [(*R*)-β^2hTyr^1]-TAPP* (**8**) sample in human plasma, taken at the start of stability testing.

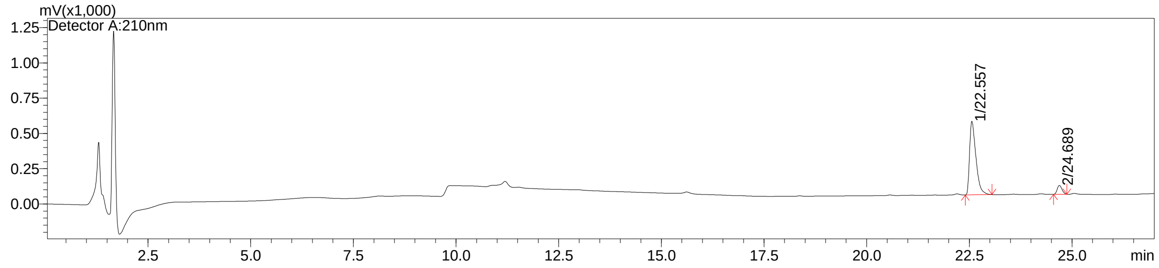

Figure SM-STAB-4. HPLC chromatogram of a [(*R*)-β^{2}hTyr^1]-TAPP* (**8**) sample in human plasma, taken at 48 h of stability testing.

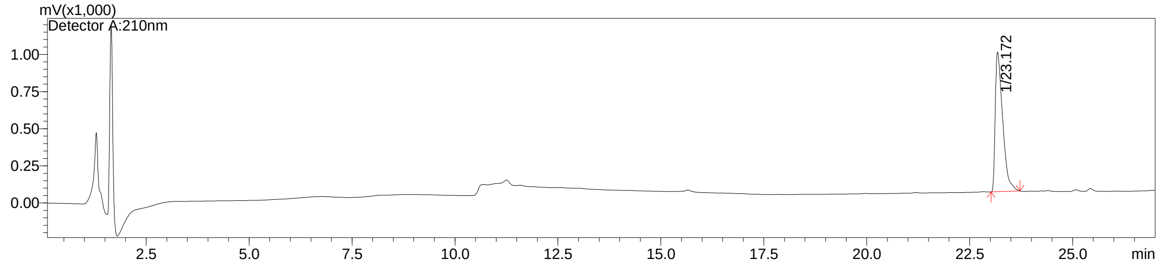

Figure SM-STAB-5. HPLC chromatogram of a [(*R*)-β^{2}h-*m*-Tyr^1]-TAPP* (**9**) sample in human plasma, taken at the start of stability testing.

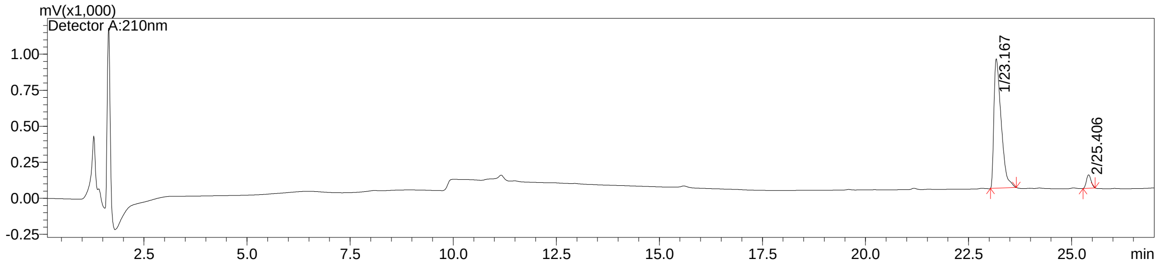

Figure SM-STAB-6. HPLC chromatogram of a [(*R*)-β^{2}h-*m*-Tyr^1]-TAPP* (**9**) sample in human plasma, taken at 48 h of stability testing.

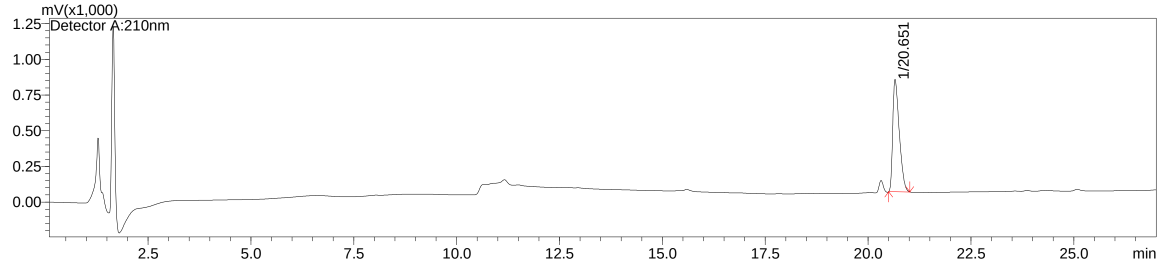

Figure SM-STAB-7. HPLC chromatogram of a [(*S*)-β^{2}hAla^2]-TAPP (**10**) sample in human plasma, taken at the start of stability testing.

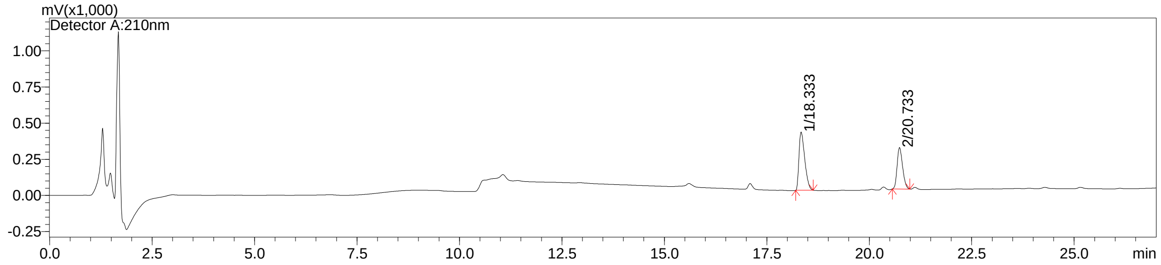

Figure SM-STAB-8. HPLC chromatogram of a [(*S*)-β^{2}hAla^2]-TAPP (**10**) sample in human plasma, taken at 4 h of stability testing.

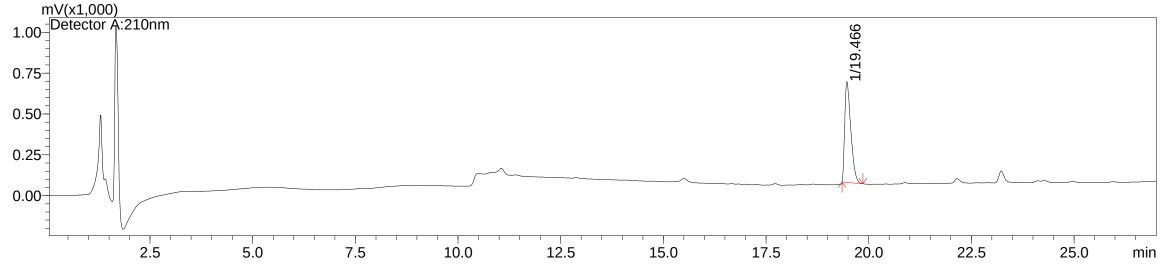

Figure SM-STAB-9. HPLC chromatogram of [(*R*)-β^{2}hPhe^3]-TAPP (**11**) sample in human plasma, taken at the start of stability testing.

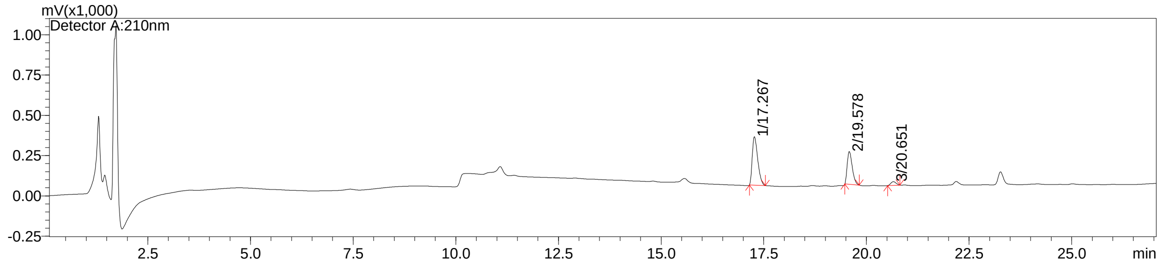

Figure SM-STAB-10. HPLC chromatogram of [(*R*)-β^{2}hPhe^3]-TAPP (**11**) sample in human plasma, taken at 48h of stability testing.

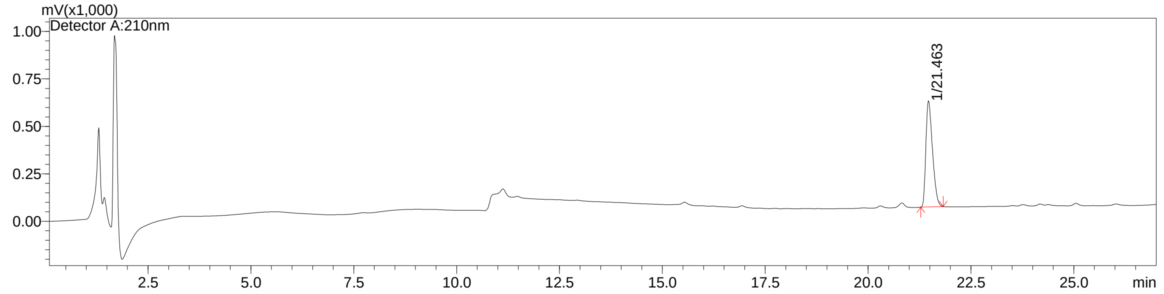

Figure SM-STAB-11. HPLC chromatogram of [(*R*)-β^{2}hPhe^4]-TAPP (**12**) sample in human plasma, taken at the start of stability testing.

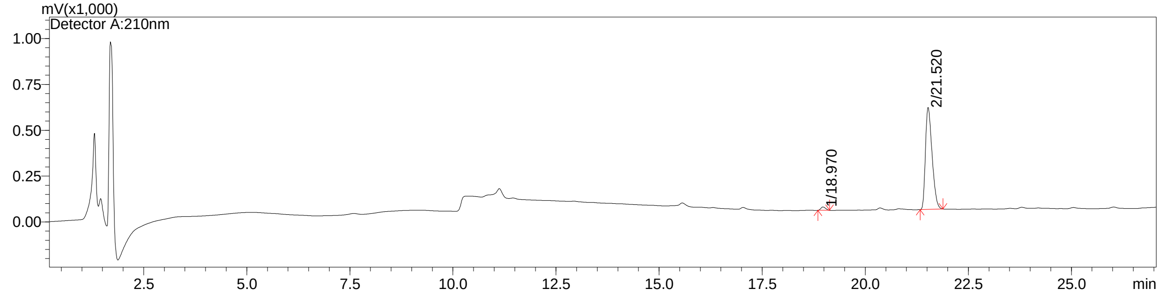

Figure SM-STAB-12. HPLC chromatogram of [(*R*)-β^{2}hPhe^4]-TAPP (**12**) sample in human plasma, taken at 48 h of stability testing.

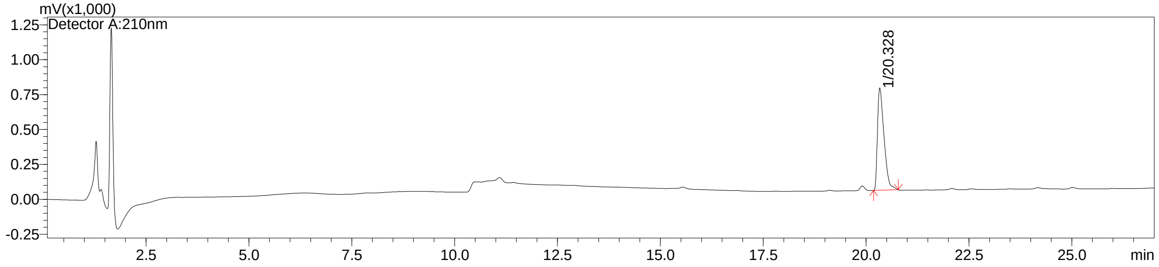

Figure SM-STAB-13. HPLC chromatogram of a [(*S*)-β^{2}hTyr^1]-TAPP* (**13**) sample in human plasma, taken at the start of stability testing.

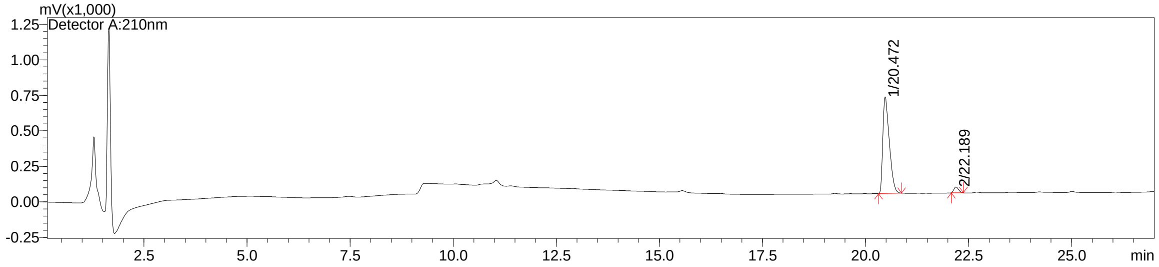

Figure SM-STAB-14. HPLC chromatogram of a [(*S*)-β^{2}hTyr^1]-TAPP* (**13**) sample in human plasma, taken at 48 h of stability testing.

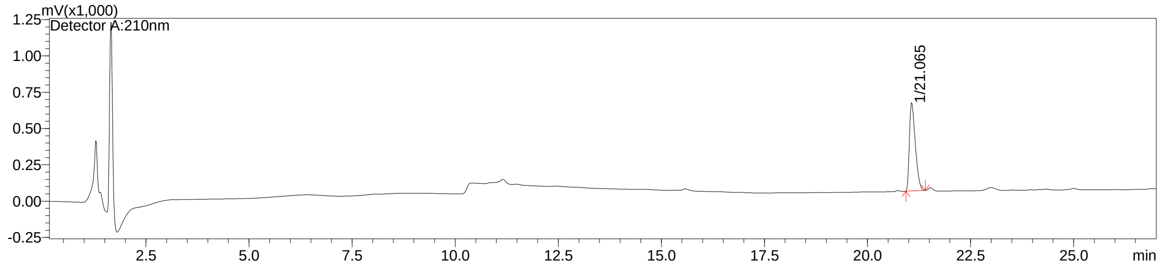

Figure SM-STAB-15. HPLC chromatogram of a [(*S*)-β^{2}h-*m*-Tyr^1]-TAPP* (**14**) sample in human plasma, taken at the start of stability testing.

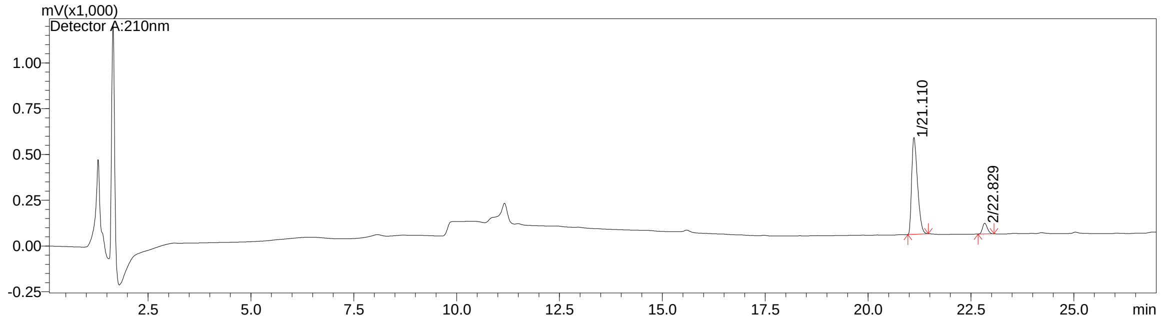

Figure SM-STAB-16. HPLC chromatogram of a [(*S*)-β^{2}h-*m*-Tyr^1]-TAPP* (**14**) sample in human plasma, taken at 48 h of stability testing.

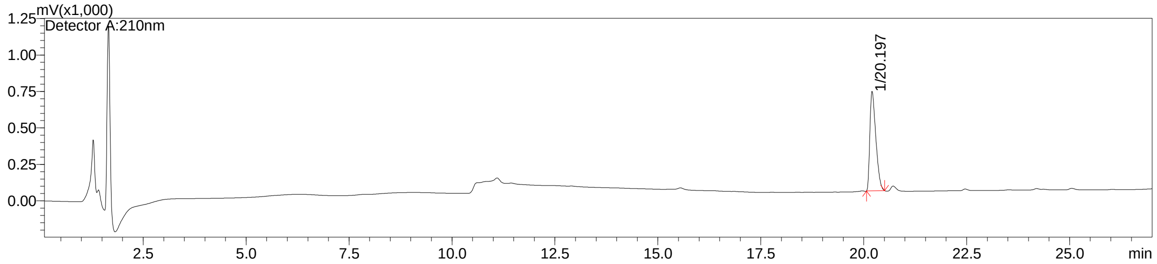

Figure SM-STAB-17. HPLC chromatogram of a [(*R*)-β^{2}hAla^2]-TAPP (**15**) sample in human plasma, taken at the start of stability testing.

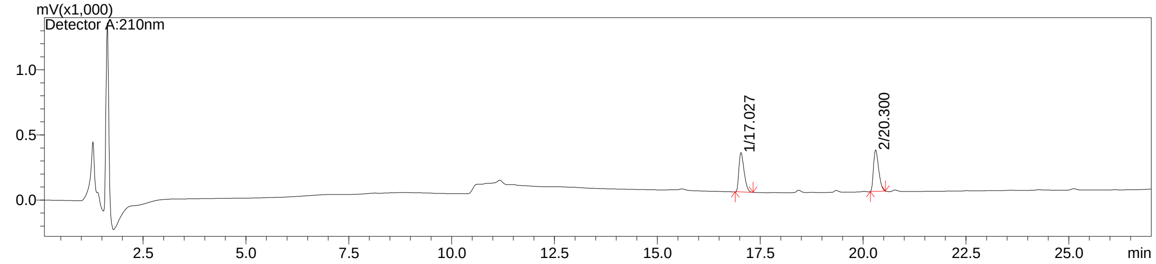

Figure SM-STAB-18. HPLC chromatogram of a [(*R*)-β^{2}hAla^2]-TAPP (**15**) sample in human plasma, taken at 4 h of stability testing.

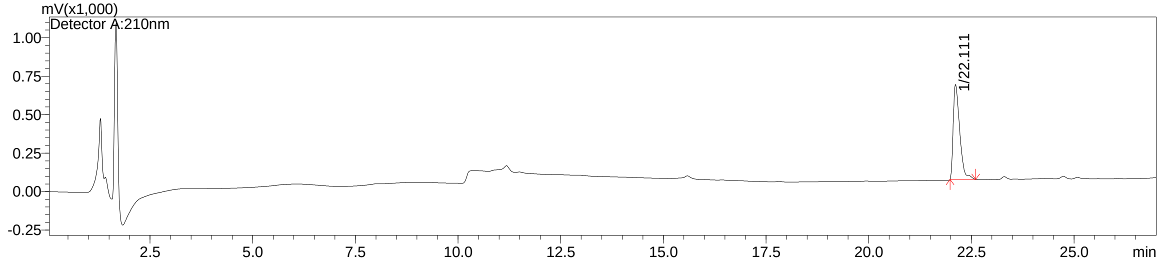

Figure SM-STAB-19. HPLC chromatogram of a [(*S*)-β^{2}hPhe^3]-TAPP (**16**) sample in human plasma, taken at the start of stability testing.

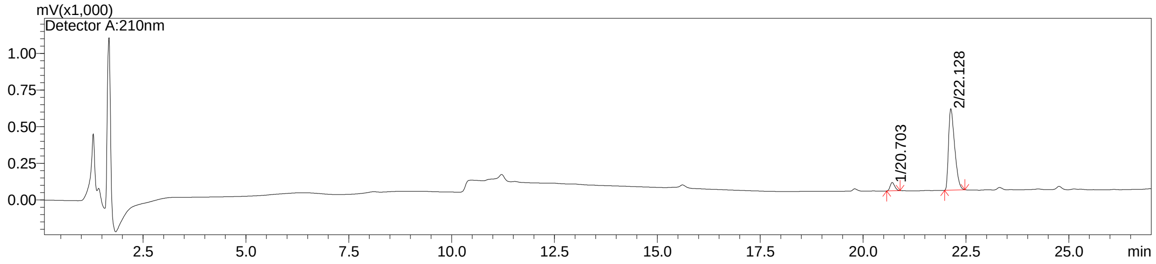

Figure SM-STAB-20. HPLC chromatogram of a [(*S*)-β^{2}hPhe^3]-TAPP (**16**) sample in human plasma, taken at 48 h of stability testing.

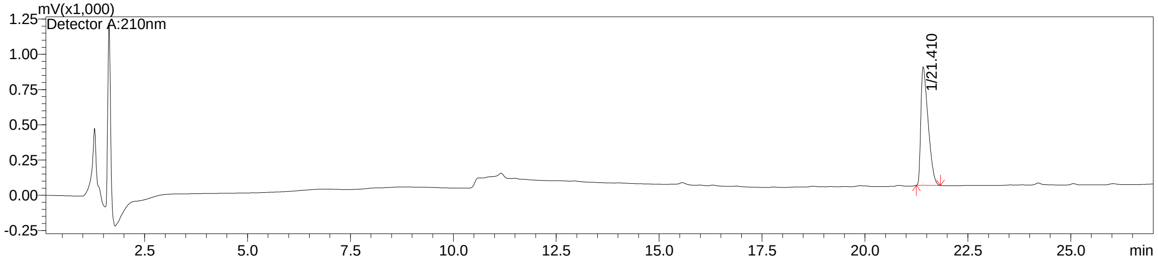

Figure SM-STAB-21. HPLC chromatogram of a [(*S*)-β^{2}hPhe^4]-TAPP (**17**) sample in human plasma, taken at the start of stability testing.

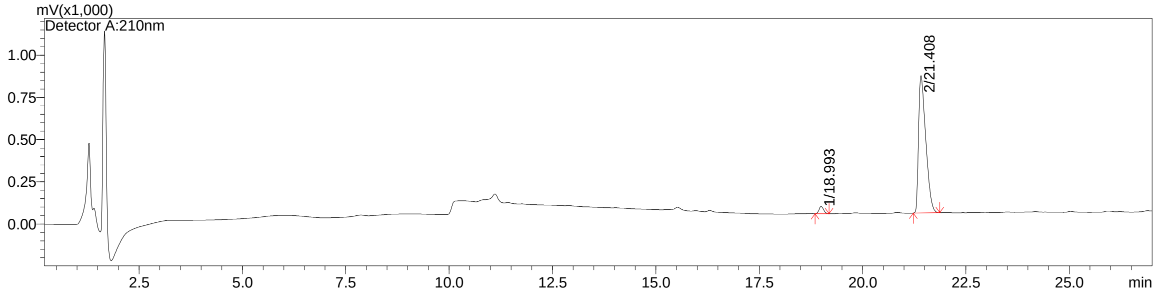

Figure SM-STAB-22. HPLC chromatogram of a [(*S*)-β^{2}hPhe^4]-TAPP (**17**) sample in human plasma, taken at 48 h of stability testing.
